# Do children’s expectations about future physical activity predict their physical activity in adulthood?

**DOI:** 10.1093/ije/dyaa131

**Published:** 2020-10-04

**Authors:** Benedetta Pongiglione, Margaret L Kern, J D Carpentieri, H Andrew Schwartz, Neelaabh Gupta, Alissa Goodman

**Affiliations:** 1 Centre for Research on Health and Social Care Management, Bocconi University, Milan, Italy; 2 UCL Institute of Education, University College London, London, UK; 3 Melbourne Graduate School of Education, University of Melbourne, Melbourne, VIC, Australia; 4 Computer Science Department, Stony Brook University, Stony Brook, NY, USA

**Keywords:** Physical activity, identity, exercise identity, life course perspective, narratives, natural language processing, latent class analysis, sociocultural context

## Abstract

**Background:**

Much of the population fails to meet recommended physical activity (PA) levels, but there remains considerable individual variation. By understanding drivers of different trajectories, interventions can be better targeted and more effective. One such driver may be a person’s physical activity identity (PAI)—the extent to which a person perceives PA as central to who they are.

**Methods:**

Using survey information and a unique body of essays written at age 11 from the National Child Development Study (*N *=* *10 500), essays mentioning PA were automatically identified using the machine learning technique support vector classification and PA trajectories were estimated using latent class analysis. Analyses tested the extent to which childhood PAI correlated with activity levels from age 23 through 55 and with trajectories across adulthood.

**Results:**

42.2% of males and 33.5% of females mentioned PA in their essays, describing active and/or passive engagement. Active PAI in childhood was correlated with higher levels of activity for men but not women, and was correlated with consistently active PA trajectories for both genders. Passive PAI was not related to PA for either gender.

**Conclusions:**

This study offers a novel approach for analysing large qualitative datasets to assess identity and behaviours. Findings suggest that at as young as 11 years old, the way a young person conceptualizes activity as part of their identity has a lasting association with behaviour. Still, an active identity may require a supportive sociocultural context to manifest in subsequent behaviour.

**Key Messages**

- Programmes targeting sedentary behaviour often succeed in increasing physical activity (PA) in the short term, but less so in the medium and long term. Using a novel machine learning approach, this study addresses the long-term associations of identity with subsequent behaviour.
- This cohort study is the first to assess the prospective association of physical activity identity (PAI)—the extent to which a person perceives PA as central to who they are and/or their future lives—expressed at age 11 with PA measured across multiple decades of adult life.
- Active PAI was predictive of PA across all adult ages (23, 33, 42, 50 and 55 years) for males but not females. Passive PAI was not predictive of PA for either genders, in models adjusted for a range of factors from birth to age 11, including self-reported PA at age 11 and a set of cognitive, social and emotional, health and socioeconomic controls in childhood.
- Considering trajectories of PA across ages 33–55, both males and females who expressed an active PAI in childhood were more likely to remain consistently active in adulthood compared with those who did not express an active PAI.
- Promoting an active PAI may improve the efficacy of policies devoted to increasing and sustaining regular physical activity.

## Introduction

Regular physical activity (PA) contributes to a range of positive physical and mental health outcomes,^1–4^ yet a large proportion of the population does not meet PA recommendations. Tracking studies find that PA decreases across adolescence, with levels and intensity continuing to decline across adulthood.^5–10^

Still, there remains considerable individual variation.^11^ PA correlates with a range of factors, including gender and age, personality, physical and mental health, social norms and customs, social support and school and neighbourhood characteristics.^12–17^ Many existing interventions focus on creating programmes, policies, structures and legislation to support, encourage and nudge people to become more active.^18^ Whereas many of these efforts have evidenced success, they are more effective for some people than for others. By understanding drivers and correlates of different trajectories, interventions can be better targeted and more effective.

From a lifespan epidemiological approach,^19^ a person’s past experiences, perceptions, cognitions and habits contribute to subsequent behaviours, including receptiveness towards and engagement in programmes and interventions. One such individual aspect may be a person’s physical activity identity (PAI), or the extent to which a person perceives PA as central to who they are.^20^ Identity includes the mindset, beliefs and interpretations that a person or group has around different behaviours, cognitions and emotions.^21^ In addition to giving meaning and value to past and current behaviour, identity can help shape expectations for the future as well as direct future behaviours.^22^

tIdentity can be measured in various ways. Most studies of PAI (sometimes expressed as ‘exercise identity’ or ‘athletic identity’) are cross-sectional and have used self-report instruments to measure PAI in adults, adolescents or children.^23–25^ Such instruments capture individuals’ consciously professed attitudes towards PA, as expressed through the completion of survey questions. As identity can be expressed through language,^21^^,^^26^ an alternative measurement approach may be to analyse texts in which individuals write about their past, present and/or projected future lives and selves.^27–32^ Through autobiographical writings, individuals consciously and unconsciously construct and present their identity, both in terms of who they are now and the ‘possible selves’^22^^,^^33^^,^^34^ they may become.

Studies find that greater PAI at one point in time correlates with concurrent higher PA levels, for both children^23^^,^^29^^,^^35^ and adults,^25^^,^^36–38^ but there is little evidence on PA identity-behaviour congruence over time, and even less so using measurement approaches other than self-report.

In the current study, we take advantage of: (i) essays written in childhood; (ii) machine learning techniques; and (iii) longitudinal data collected from a large, nationally representative cohort across five decades, to examine the extent to which PAI expressed in childhood predicts PA levels and PA trajectories across adulthood.

## Methods

### Participants

Participants were drawn from the National Child Development Study (NCDS), a UK-based study that has followed a cohort of over 17 000 individuals prospectively across their lives. In 1958, 98.1% of all babies born within England, Scotland and Wales in the first week of March were included in the original sample. Subsequent assessments occurred at multiple occasions throughout childhood and adult life up to age 55, and comprised a broad range of topics including parental background, social class, physical and mental health, cognition, emotional and behavioural issues, education, economic circumstances, employment, health-related behaviours, family life, attitudes and social participation.

In 1969, the assessment invited the 11-year old cohort members to spend 30 min responding to the question: ‘Imagine you are now 25 years old. Write about the life you are leading, your interests, your home life, and your work at the age of 25’. Of the original sample, 14 757 completed the year 11 survey, and 13 669 responded to the essay prompt.^39^^,^^40^ We were able to transcribe 10 567 surveys. Missing surveys were due to poor quality of the microfiche (a flat piece of film that preserves images of old documents) or missing essays. Transcriptions included spelling mistakes, and identifying details were replaced as <name> for names of individuals or <xxxx> for other identifying information (e.g. an address). The final transcribed essays can be accessed through the UK Data Service.^41^

Due to missing data on the adult measures, our main analyses predicting future activity levels included 8866 participants (49.5% females) who had essay information available and at least one adult PA outcome from the ages 23–55 surveys, and were alive and not emigrated by age 55 (see Supplement S1, available as Supplementary data at *IJE* online for participant flow). For PA trajectories, we included 8158 participants (50% females) with at least one PA observation from age 33 to 55.

### Measures

Making use of the rich NCDS dataset, measures used in the current study included: the essays; engagement in PA at ages 23, 33, 42, 50 and 55; and control variables from assessments at birth and ages 7 and 11.

### Linguistic indicators of physical activity identity using machine learning techniques

Our primary predictor was PAI at age 11, which we automatically derived from the essays using machine learning techniques. We operationalized PAI as writing about PA pastimes as an adult (e.g. ‘My biggest interest will still be swimming’, ‘On Sundays the 3 of us go horse riding’). Rather than manually code all 10 567 essays, machine learning techniques enabled us to code a subset of essays (i.e. ‘training set’), and then build a machine classification model based on those ratings to recognize similar cases in the remaining essays in a time and resource-efficient manner. Alternatively, we could manually create lexica indicative of active or spectator activity, and then count how often words in the lexica occur. However, words without their context are ambiguous (see Schwartz *et al*.^42^ for an error analysis of manual lexica). Learning from a corpus of real examples enables the machine learning model to distinguish words that reliably capture the correct context, such that machine learning classifiers have been found to be much less error-prone for content classification.^43^^,^^44^ To verify our application, we compared our machine learning classifier with a manual lexicon and found that the machine learning classifier had much higher specificity and sensitivity (see Supplement S3, available as Supplementary data at *IJE* online).

We first trained the classification model to distinguish whether a person wrote about activities as a participant (i.e. directly engaging in the activity) or as a spectator (i.e. watching others engage in sport, e.g. ‘We're at Loftes Road stadium were the Rangers play and they're playing Portmouth’). Whereas in both cases, activity mattered enough to the child to include it in a short essay, we would expect that mentioning active participation would more likely relate to subsequent engagement in active behaviours. To create the training set, we first randomly selected 500 essays, and two of the authors (B.P. and M.L.K.) rated any mention of activity (0 = not mentioned, 1 = one or more activities mentioned) as ‘active’ (indicates participating in moderate or vigorous PA, representing PAI) and/or ‘spectator’ (indicates watching or playing a passive role in activities). Inter-rater agreement (Cohen’s kappa) was κ = 0.90 for active mentions and κ = 0.82 for spectator mentions, indicating substantial agreement.^45^ Full agreement was reached through discussion.

The 500 coded essays were then used to train a classification model that recognized mentions of active and spectator activity. Specifically, we used the machine learning technique support vector classifier (SVM),^46^^,^^47^ which has been successful with similar text categorization tasks,^48–50^ and aligns with other popular classification models such as random forests, penalized logistic regression and ensemble techniques.^44^^,^^51^ As input to the model, we used one to three n-grams (i.e. one-, two- or three-word phrases), filtered to those mentioned in at least 2% of essays (10 576 distinct n-grams). Rather than encode the features as relative frequency counts, which are unstable for short documents,^44^ we encoded the features as binary (0 = does not exist, 1 = does exist). This helps the classifier identify multiple types of evidence (i.e. phrases) related to activity, and it has previously worked well for similar tasks.^43^ An L_1_ regularization penalty (‘the Lasso’) and linear kernel was used with the SVM, which is known to help avoid overfitting the model when the number of features (in this case 839) is greater than the number of observations (in this case 500).^52^ The specific penalty value was set based on the training data during the cross-validation process.

To assess classification accuracy, we used 10-fold cross-validation,^53^ in which we split our sample into 10 equal-sized, non-overlapping, stratified partitions, and then fitted the models (also setting the regularization penalty constant) over nine partitions (i.e. the training set), and then tested the model fit on the remaining held-out partition (i.e. the test set). The classifier model achieved accuracy levels of 74.5% for active mentions and 87.2% for spectator mentions. We also assessed the classifier performance using the receiver operating characteristic (ROC) curve,^54^ which resulted in area under the curve statistics of 0.838 for active mentions and 0.754 for passive mentions (see Supplement S3 for ROC curves). Key predictive words indicating active status included swimming, football, play, horse, team, swim, dance, tennis, riding and footballer, suggesting face validity for the status predictions*.* The classifier was then applied to the remaining essays, which gave each essay a 0 or 1 indicator score on predicted active and spectator variables. All machine learning analyses were performed with the Python package, Differential Language Analysis ToolKit^55^ (see Supplement S7 for code).

### Self-reported physical activity in childhood

At age 11, participants’ mothers indicated whether or not the child participated in sport out of school (1 = hardly ever, 2 = sometimes, 3 = most days).

### Self-reported physical activity in adulthood

As a long-running study that was not designed to study PA, gold-standard measures of PA were not available, but some activity information was included in the surveys. At age 23, participants indicated how often they played sports of any kind in the past 4 weeks, which was coded as at least once a week (1) or less than once a week (0). At ages 33, 42, 50 and 55, participants indicated their frequency of regularly exercising. We created a dichotomous code for each measurement occasion, indicating exercising at least once a week (1) or less than once a week (0).

As the age 23 question differed from later time points, we included all five time points to consider PA levels but excluded the age 23 question for PA trajectories. PA trajectories were estimated using the age 33–55 dichotomized variables through latent class analysis (LCA),^56^ separately by gender.

**Figure 1:**
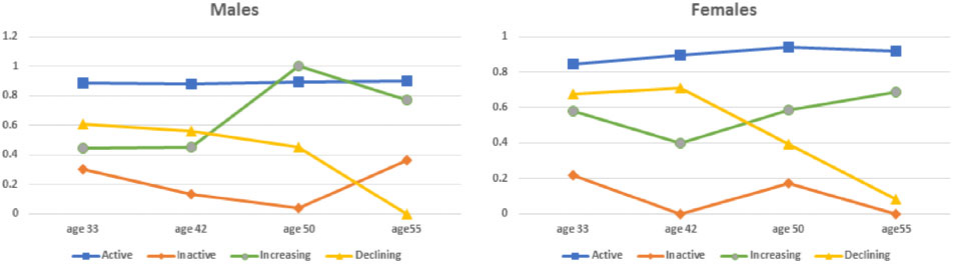
Physical activity (PA) trajectories from age 33 through age 55, 4-class model, separately by gender

As illustrated in Figure 1, we selected a four-class model, based on indicators of fit and interpretation of results (i.e. interpretation of classes) (see Supplement S4, available as Supplementary data at *IJE* online for analysis details and trajectories based on 2-, 3-, 5- and 6-class models).

### Control variables

An advantage of the NCDS is the large number of survey variables that can be controlled in analyses. We included sociodemographic, health, functional and behavioural variables, based on the birth and year 11 assessments, which are known to be associated with PA in adolescence and adult life.^57–59^ These include: birthweight; whether the mother smoked during pregnancy; father’s social class; child’s body mass index; enuresis measured as whether the child is completely dry at night and during the day; poor physical coordination as reported by parents; an indicator of the child’s behaviour in the school setting, measured using the Bristol Social Adjustment Guides; an index of behaviour difficulties in the child captured by the Rutter scale^60^; general ability test score; and two indicators of childhood health (time off school for ill health and number of times the respondents was admitted to hospital by age 11). We also included child’s self-reported PA at age 11, as described above.

### Data analysis

Using logistic regression, the two dichotomous indicators of active and spectator PAI were used separately to predict the self-reported binary measures of PA participation at each measurement occasion. Multinomial logistic regression^61^^,^^62^ was used to predict the probabilities of the different outcomes of the categorical latent measure of PA trajectory in adulthood. We performed these analyses in two steps, first estimating PA class with LCA, and then adding the PA class variable in the logistic regression model as an ‘observed’ dependent variable, separately for men and women. The application of LCA to repeated measures is often referred to as longitudinal LCA (LLCA)^63^ and enables identification of common patterns of discontinuous development in a categorical manifest or latent variable. The latent class model assumes that any covariation among indicators of the outcome is accounted for by the latent class variable (i.e. the assumption of local independence^64^). LLCA requires fewer assumptions than generalized linear models such as growth mixed models and latent class growth analysis (LCGA),^63^ and LLCA models patterns of states across time rather than modelling scaled change, aligned with our interests here. As a sensitivity analysis, we estimated trajectories of physical activity using LCGA, which takes advantage of time-ordered outcomes (see Supplement S5, available as Supplementary data at *IJE* online). However, results were unstable when covariates were included in the model, and we retained LLCA for our analyses.

The two-step estimation fails to account for each person’s class probability, such that it can be more biased than simultaneous estimation. However, with simultaneous estimation, the latent classes are not directly comparable between the active and spectator models and the distribution of participants across the four classes differs, making their interpretation less interpretable. As a sensitivity analysis, we also simultaneously performed the LLCA and multinomial model, which accounts for each person’s class probabilities, finding similar results (see Supplement S6, available as Supplementary data at *IJE* online).

Missingness was assumed to be random (MAR)^65^ and addressed using multiple imputation (MI) with chained equations. Full information maximum likelihood (FIML) estimation was used to estimate PA trajectory in MPlus (version 8.1); trajectories were then regressed on PAI and variable controls using the imputed dataset (see Supplement S2 for approach descriptionand Supplement S7 for analysis codes, available as Supplementary data at IJE online). Both MI and FIML provide unbiased estimates in the presence of missing data under the MAR assumption.^65^^,^^66^

As an additional supplemental analysis (see Supplement S8, available as Supplementary data at *IJE* online ), we also used MI to impute values for all individuals in the full dataset who did not die or emigrate by age 50 (*N *=* *15 806). Findings were mostly consistent with those reported below, where we restored observations only for those who completed the essay at age 11 and reported at least one measure of PA in adulthood (*N *= 8866).

### Results

#### Descriptives

Using the automatic classifier, 20.6% of males were classified as active, 15.7% were active and spectators (i.e. they mentioned both engaging in and watching others engage in PA) and 5.9% were spectators only. Among females, 28.1% were classified as active, 2.2% were active and spectators and 3.2% were spectators only; 57.8% of males and 66.5% of females did not mention PA in their childhood essays.

In the age 11 survey, 54% of boys and 37% of girls reported engaging in sport most days. Both active and spectator PAIs were associated with sport participation (active PAI: Spearman ρ = 0.107; spectator PAI: Spearman ρ = 0.119).

Table 1 summarizes activity across adulthood. At age 23, 42% of males and 22% of females reported playing sports at least once a week, with males more likely to play sports than females (1df-chi square = 398, *P*-value <0.001). Across ages 33–55, about two-thirds of respondents reported exercising at least once a week, with no differences between genders.

**Table 1:** Proportion (95% CI) of participants who reported playing sports (age 23) or exercising (ages 33–55) at least once a week, by gender and assessment occasion

	Males	Females	*P*-value
Age 23	42.9 (41.4; 44.5)	21.5 (20.2; 22.8)	<0.001
Age 33	68.1 (66.6; 69.7)	69.8 (68.3; 71.3)	0.131
Age 42	64.8 (63.2; 66.4)	66 (64.5; 67.6)	0.281
Age 50	70.7 (69.1; 72.3)	68.9 (67.3; 70.5)	0.119
Age 55	63.1 (61.3; 64.8)	64.1 (62.4; 65.8)	0.403

#### Child physical activity identity predicting adult physical activity

**Figure 2:**
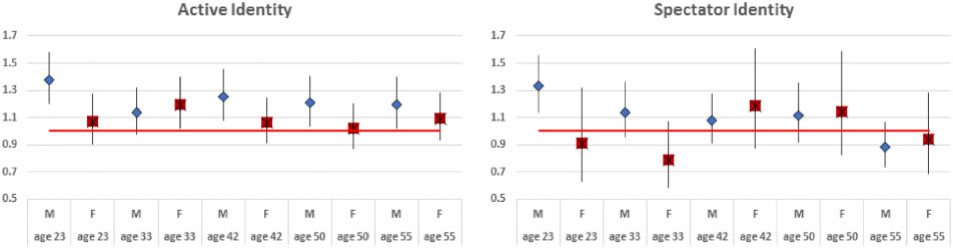
Odds ratios with 95% confidence intervals predicting adult activity from childhood active and spectator identities, controlling for self-reported physical activity at age 11, family background and physical and mental health, separately by gender

As illustrated in Figure 2, active PAI was predictive of subsequent activity for males at each time point except age 33, but for females it was only predictive at age 33. Spectator identity predicted greater participation in sports at age 23 for males but was not predictive of subsequent activity involvement. Spectator identity was not predictive for females.

The effect sizes in Figure 2 are based upon the fully adjusted model. Compared with the univariate model, effect sizes only slightly declined when the control variables were added. Results remain consistent whether or not childhood self-reported PA was included, suggesting that identity (childhood PAI) and behaviour (self-reported childhood PA) predict unique aspects of adult PA (see Supplement S8 for full model results, and for comparisons among the univariate model, a model excluding childhood PA and the fully adjusted model).

For PA trajectories from age 33 to age 55, we selected a four-class model: ‘always active’ (60% males, 49% females); ‘always inactive’ (16% males, 6% females); ‘fluctuating/increasing PA’ (5% males, 26% females); and ‘declining PA’ (19% males, 19% females) (Figure 1). Table 2 reports results of the multinomial logistic regression as relative risk ratios (RRRs). For males, active PAI predicted lower risk of belonging to the ‘never active’ [RRR = 0.656, 95% confidence interval (CI) 0.542;0.802] and ‘declining PA’ clusters (RRR = 0.803, 95% CI 0.675; 0.957) than ‘always active’. Thus those who expressed an active PAI compared with those who did not had almost a 35% lower risk of never being active in adulthood rather than being always active, and 20% lower risk of having ‘declining PA’ in adulthood rather than being always active. For females, active PAI predicted lower risk of ‘fluctuating/increasing PA’ (RRR = 0.774, 95% CI 0.652; 0.918) or ‘declining PA’ (RRR = 0.842, 95% CI 0.699; 1.015) than being ‘always active’. Spectator PAI was not predictive of PA trajectory. Results remained the same after controlling for childhood self-reported PA; PAI and self-reported PA independently predicted PA trajectories in adulthood (see Supplement S9, available as Supplementary data at *IJE* online).

**Table 2:** Relative risk ratios (RRR) for the fully adjusted model of physical activity (PA) trajectory classes compared with baseline trajectory class ‘always active’, separately by gender

	Male			Female		
	Fluctuating/increasing PA	Declining PA	Always inactive	Fluctuating/increasing PA	Declining PA	Always inactive
Active PA identity	0.85	0.803^**^	0.659^***^	0.774^***^	0.842^*^	1.036
	(0.621; 1.163)	(0.675;0.957)	(0.542–0.802)	(0.652; 0.918)	(0.699;1.015)	(0.769;1.395)
Spectator PA identity	0.889	1.157	0.794^*^	0.828	0.873	1.127
	(0.612; 1.292)	(0.952;1.407)	(0.631–1.000)	(0.585; 1.173)	(0.597;1.278)	(0.642;1.978)

Reference group is no mention of active or spectator activities in the childhood essays. 95% CI in parentheses.

* *P* <0.1;

** *P* <0.05;

*** *P* <0.01.

### Discussion

Combining narrative information about envisioned futures, natural language processing techniques and survey data collected prospectively across five decades from a large nationally representative sample, we tested the extent to which PAI in childhood predicted subsequent engagement in physical activity across adulthood. Active PAI, which we operationalized as writing about oneself engaging in active pastimes, was correlated with greater activity participation at each age for males but not for females, and was correlated with PA trajectory from age 33 through age 55 for both genders. Spectator PAI, although related to childhood PA, was not related to PA in adulthood.

An ongoing challenge is how to encourage people to remain active across life. Although many programmes successfully increase activity for a short period of time, studies suggest that it is ongoing, habitual PA that promotes good physical and mental health outcomes.^1^^,^^3^^,^^11^^,^^67^ Some individuals are naturally more drawn towards being active than others. For instance, a meta-analysis of 35 samples found that individuals lower in neuroticism and higher in extraversion or conscientiousness were more likely to be active.^16^ Self-efficacy and agency, goals and motivation, self-esteem and intentions affect one’s behaviours.^68–71^

The current work suggests that PAI may be an important driver of ongoing PA behaviour. A number of studies have found evidence for identity-behaviour congruence in the PA domain,^24^^,^^35^^,^^69^ supporting the hypothesis that PA identity and behaviour are concurrently correlated. We draw a similar conclusion based on the measure of age 11 physical activity, which was assessed concurrently with the age 11 essay-writing exercise. Identity theory also suggests that even though identity is changeable over time, it contains a strong degree of continuity,^72^ suggesting that early-life PAI should correlate with PA later in life—i.e. that PA identity-behaviour congruence is a longitudinal phenomenon. To the best of our knowledge, this is the first study to consider the prospective associations of PAI in early adolescence on PA behaviour across multiple decades of adult life.

Our study is also unique in its use of written essays, in which individuals look forward from one stage of life (childhood) to another (adulthood), rather than using self-report instruments to measure PAI. Future-oriented biographical writings require individuals to construct and present an identity that builds on their current lives and self-understanding while also accounting for their imagined future selves.^40^ The construction of a congruent longitudinal identity may be more feasible when writing about leisure interests and activities such as swimming or football, as such activities can be experienced during both childhood and adulthood, in contrast to adult-specific activities, such as employment, that are unknown life events at early adolescence.

It is interesting to note that active PAI was related to adult PA at single time points in the univariate analysis for females (see Supplement S8), but the association was confounded by childhood background in a way that was not observed for males. One potential reason for the lack of independent association of PAI among females is the social nature of identity.^21^ Boys were more physically active than girls according to the parental report in the age 11 (actual PA), and made more mentions of PA in the essays, for both active and spectator activities (PAI). At the time these essays were written (1969), it may have been considered more appropriate for males to engage in leisure-time PA than females.^73^ Indeed, in the 1960s, playing and watching sport remained far more popular among men, despite significant advances in female participation rates and the profiles of some leading sportswomen.^74^ It is also likely that there are gender differences in the essays in the specific activities mentioned (e.g. football versus netball), and future studies might consider whether specific activities reported within the essays are differentially related to future outcomes.

Interestingly, when we looked at how PAI was related to PA trajectories over adult life rather than at single time points, active PAI predicted an active trajectory for females as well as males. Table 1 suggests that PA has become less gendered over time. It is possible that PAI was predictive of behaviour when the context was supportive (generally in the case of men), but less predictive of behaviour when the context was unsupportive (in the case of many women, leading to inconsistent results). Regardless of whether patterns are due to the period, age or cohort effects, the pattern of results implies that PAI development and maintenance need to be supported by social norms and environmental contexts that encourage people of all backgrounds to be active, and to see PA as an important part of who they are now and who they will be in the future. Many interventions focus on creating programmes and structures or using external rewards to motivate behaviour. However, promoting PA may not simply be about the behaviour itself, but also involve the salience and importance of PA to identity^75^—factors which are influenced not just by ‘internal’ traits but also by social and cultural norms.

Our study used information from a long-running study that captured information on PA alongside many other domains. The measures of PA are likely to suffer from the biases typical of self-reported measures and imprecise measurement. Although questions and single-choice answers were asked consistently across sweeps, intensity and duration were unavailable. We did not distinguish types of adult physical activity; some activities might have shown stronger associations with PAI than others. Our approach to measuring PAI is novel in that it draws on children’s essays about their projected future lives and uses machine learning techniques to analyse those essays, but the automatic detection of the PAI indicators may understate mentions of physical activity within the writing, potentially attenuating results. The validity of this approach needs to be further tested in other studies, using other samples and types of text. Although our models adjust for a rich set of survey controls covering social emotional, cognitive, health and socioeconomic domains, we cannot fully rule out that other unobserved factors correlated both with PAI and adult PAI may be partly driving the associations found. Finally, analyses are correlational in nature, and although there is a temporal ordering between the childhood and adult measures, we cannot ascertain causation.

Our study suggests that the use of machine learning techniques to analyse large qualitative datasets can play a meaningful role in assessing the association between people’s perceptions of themselves and their subsequent behaviours. Findings raise the intriguing possibility that the way that a person conceptualizes activity as part of their identity—or not—can have a lasting impact on behaviour.

### Supplementary data

Supplementary data are available at *IJE* online.

### Funding

This work was supported by the Economic and Social Research Council (reference ES/N00650X/1 and ES/M008584/1). We also thank ESRC for its support of the National Child Development Study and the wider activities of the Centre for Longitudinal Studies, through the CLS Resource Centre 2015–2020 (reference ES/M001660/1).

### Conflict of interest

None declared.

## Supplementary Material

dyaa131_supplementary_data
