# Supplementary material for: Do children’s expectations about future physical activity predict their physical activity in adulthood?
Source: Int J Epidemiol. 2020 Oct 4;49(5):1749–58. doi: 10.1093/ije/dyaa131 (PMC7746399; doi:10.1093/ije/dyaa131)
Supplement: dyaa131_supplementary_data [file dyaa131_supplementary_data.docx]

**Do Children’s Expectations about Future Physical Activity**

**Predict their Physical Activity in Adulthood?**

**Supplemental Material**

Benedetta Pongiglione ^1,2^, Margaret L. Kern ^3^, JD Carpentieri ^2^,

H. Andrew Schwartz ^4^, Neelaabh Gupta ^4^, Alissa Goodman ^2^,

^1^ Bocconi University Centre for Research on Health and Social Care Management, ^2^ University College London Institute of Education, ^3^ University of Melbourne, ^4^ Stony Brook University,

Please address correspondence to Peggy.Kern@unimelb.edu.au

Table of Contents

[S1. Flow of participants, resulting in final sample included in the study 4](#_Toc26292016)

[S2. Multiple Imputation Approach 5](#_Toc26292017)

[S3. Machine Learning Classifier 7](#_Toc26292018)

[Assessment of classification accuracy using the ROC curve 7](#_Toc26292019)

[Machine Learning Classifier Versus Manual Lexicon Approach Comparison 8](#_Toc26292020)

[S4. Physical Activity Trajectory Estimation Selection Process 9](#_Toc26292021)

[Latent class model goodness of fit indicators by gender 9](#_Toc26292022)

[Trajectories of physical activity from age 33 through age 55, based on 2, 3, 5 and 6-class models 9](#_Toc26292023)

[S5. Latent Class Growth Analysis 11](#_Toc26292024)

[Latent class model goodness of fit indicators by gender 11](#_Toc26292025)

[Trajectories of physical activity based on one-step model estimation, age 33-55, 4-class models 12](#_Toc26292026)

[S6. Simultaneous estimation of LCA and multinomial logistic regression model 13](#_Toc26292027)

[Latent class model goodness of fit indicators by gender 13](#_Toc26292028)

[Trajectories of physical activity based on one-step model estimation, age 33-55, 4-class models 13](#_Toc26292029)

[Relative Risk Ratios (RRR) for the fully-adjusted model of PA trajectory classes compared to baseline trajectory class “always active”, separately by gender, one step. 15](#_Toc26292030)

[S7. Analytic Codes 16](#_Toc26292031)

[Stata Analysis Code 16](#_Toc26292032)

[Mplus Analysis Code 23](#_Toc26292033)

[Differential Language Analysis ToolKit (DLATK) Code 26](#_Toc26292034)

[S8. Odd ratios predicting adult activity from childhood active and passive PAI 28](#_Toc26292035)

[Odds ratios predicting adult activity from childhood active PAI, males. 28](#_Toc26292036)

[Odds ratios predicting adult activity from childhood passive PAI, males. 29](#_Toc26292037)

[Odds ratios predicting adult activity from childhood active PAI, females. 30](#_Toc26292038)

[Odds ratios predicting adult activity from childhood passive PAI, females. 31](#_Toc26292039)

[Odds ratios predicting adult activity from childhood active and passive PAI, obtained from univariate and adjusted models. Males 32](#_Toc26292040)

[Odds ratios predicting adult activity from childhood active and passive PAI, obtained from univariate and adjusted models. Females 33](#_Toc26292041)

[Odds ratios predicting adult activity from childhood active PAI, males, N=8,042 34](#_Toc26292042)

[Odds ratios predicting adult activity from childhood passive PAI, males, N=8,042 35](#_Toc26292043)

[Odds ratios predicting adult activity from childhood active PAI, females, N=7,764 36](#_Toc26292044)

[Odds ratios predicting adult activity from childhood passive PAI, females, N=7,764 37](#_Toc26292045)

[S9. Fully adjusted relative risk models 38](#_Toc26292046)

[Relative Risk Ratios (RRR) of active PAI for fully-adjusted model of PA trajectory classes compared to baseline trajectory class “always active”. Males. 38](#_Toc26292047)

[Relative Risk Ratios (RRR) of spectator PAI for fully-adjusted model of PA trajectory classes compared to baseline trajectory class “always active”. Males. 39](#_Toc26292048)

[Relative Risk Ratios (RRR) of active PAI for fully-adjusted model of PA trajectory classes compared to baseline trajectory class “always active”. Females. 40](#_Toc26292049)

[Relative Risk Ratios (RRR) of spectator PAI for fully-adjusted model of PA trajectory classes compared to baseline trajectory class “always active”. Females. 41](#_Toc26292050)

# S1. Flow of participants, resulting in final sample included in the study


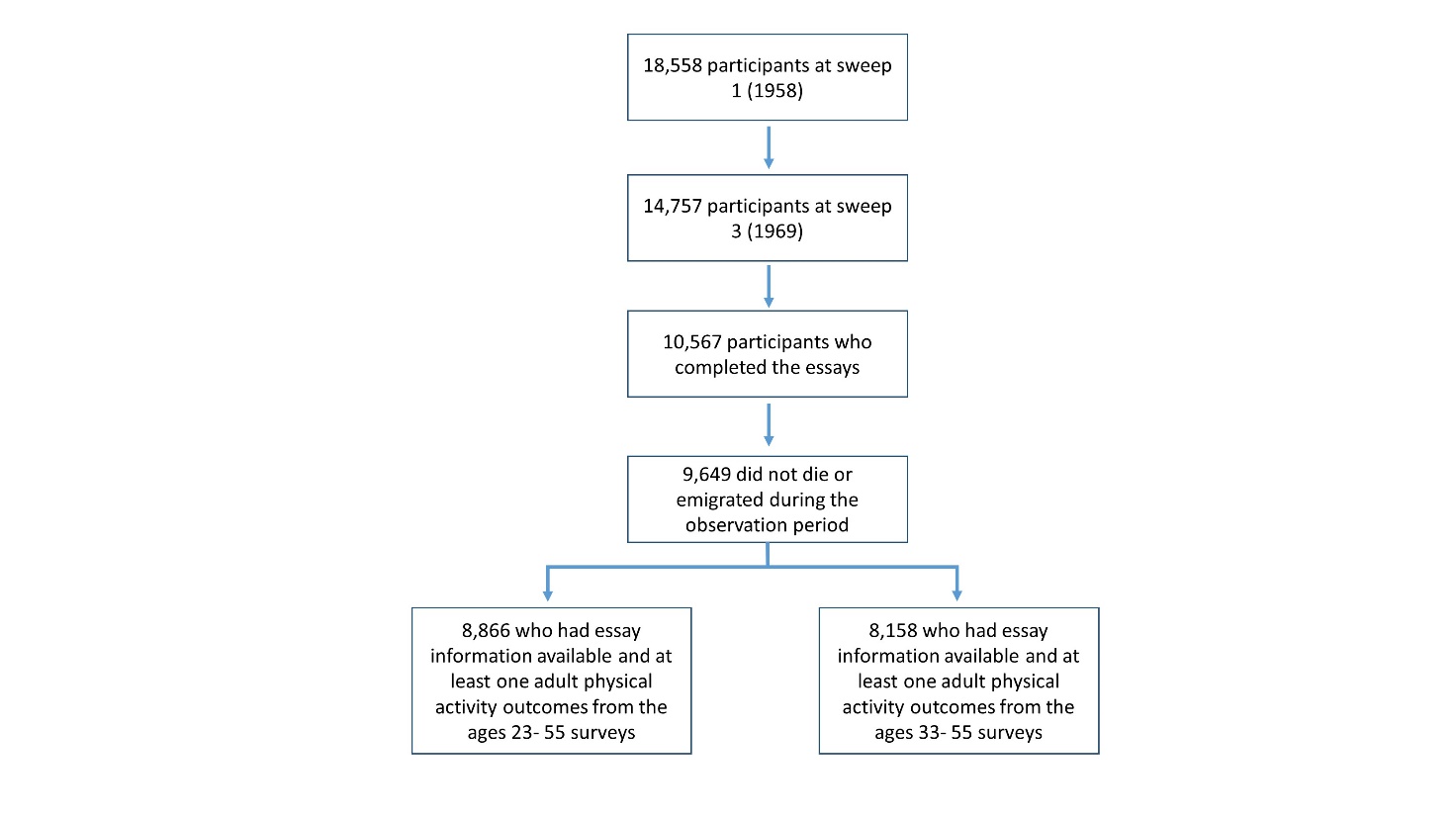


# S2. Multiple Imputation Approach

The implementation of the multiple imputation (MI) approach was based on the missing data strategy that has been developed at the Centre for Longitudinal Study (CLS)^[[1]](#footnote-1)^, which is home to the National Child Development Study (NCDS), as a systematic data-driven approach to identifying predictors of non-response. The strategy robustly identifies predictors of missingness specifically within the NCDS sample and provides a validated stepwise approach for implementation in specific studies.

The predictors of response (at any point in time) were identified by the CLS data-driven missing data strategy, which considers variables that are part of the model (i.e., outcome(s), exposures, and controls) and auxiliary variables (i.e., variables whose sole purpose is to improve the performance of the missing data methods, even when they are not relevant to the hypotheses of scientific interest.^[[2]](#footnote-2)^ Among auxiliary variables, these include: (1) a set of four NCDS variables measured at birth that have complete records and have been found to be strong predictors of missingness (see explanation below); (2) a key predictor of the outcome (which is not necessarily a confounder) (e.g., sport at age 11 predicting PA at ages 23 to 55); (3) auxiliary variables that are associated with the outcome; and (4) the strongest predictor of responses at each age considered.

The initial 1958 NCDS sample consisted of 17,415 individuals who have been followed across 10 sweeps from birth until age 55. A total of 17,412 variables have been deposited in the UK Data Service. Potentially, all variables could be used as predictors of non-response, but this would create unwieldy analyses. To reduce bias, a data-driven approach is used to reduce the number of predictors included. First, the “routed” variables (i.e., variables where a response is dependent on a previous question and only cohort members that gave a specific response are asked these questions) are excluded, which avoids sample selection. Second, binary variables with prevalence less of than 1% and variables with item non response > 50% are excluded. Then, summary scores are calculated for all scales, and if waves include multiple scales assessing the same construct, only the scale repeated across the most waves is chosen. This resulted in 616 variables, including both self-reported measures and biomarkers. In addition, a “propensity to respond” summary that captures the number of participants that took part to all NCDS sweeps was calculated.

A binary non-response variable was created for each wave of NCDS from age 7 onwards. Non-response was defined as no participation in the survey either because of refusal, the survey team not been being able to establish contact, or because contact was not attempted. Mortality and emigration were not considered non-response.

Predictors of non-response were identified for each wave employing a two-step analytic strategy. A series of within wave multivariable regressions with wave specific non-response as the outcome was estimated for each wave starting from age 7. Non-response was modelled with a log binomial model with robust standard errors. Variables whose association with non-response reached the conventional 5% level of statistical significance were retained to be used at stage 2. At stage 1, the Least Absolute Shrinkage and Selection Operator (LASSO) was also employed as a robustness check for variable selection, that produced very similar results with log binomial regressions.

In the second step of the analysis, these variables were simultaneously entered into the models. As a variable selection criterion, p < 0.001 was used. Consistent predictors of non-response variables were defined as associated with non-response in the same direction in more than 2 sweeps of NCDS.

# S3. Machine Learning Classifier

## Assessment of classification accuracy using the ROC curve

As a further assessment of classification accuracy, we assessed the classifier performance using the Receiver Operating Characteristic curve (ROC), which resulted in area under the curve (AUC) statistics of 0.838 for active mentions and 0.754 for passive mentions. Below we show the ROC curves for active and passive activity.


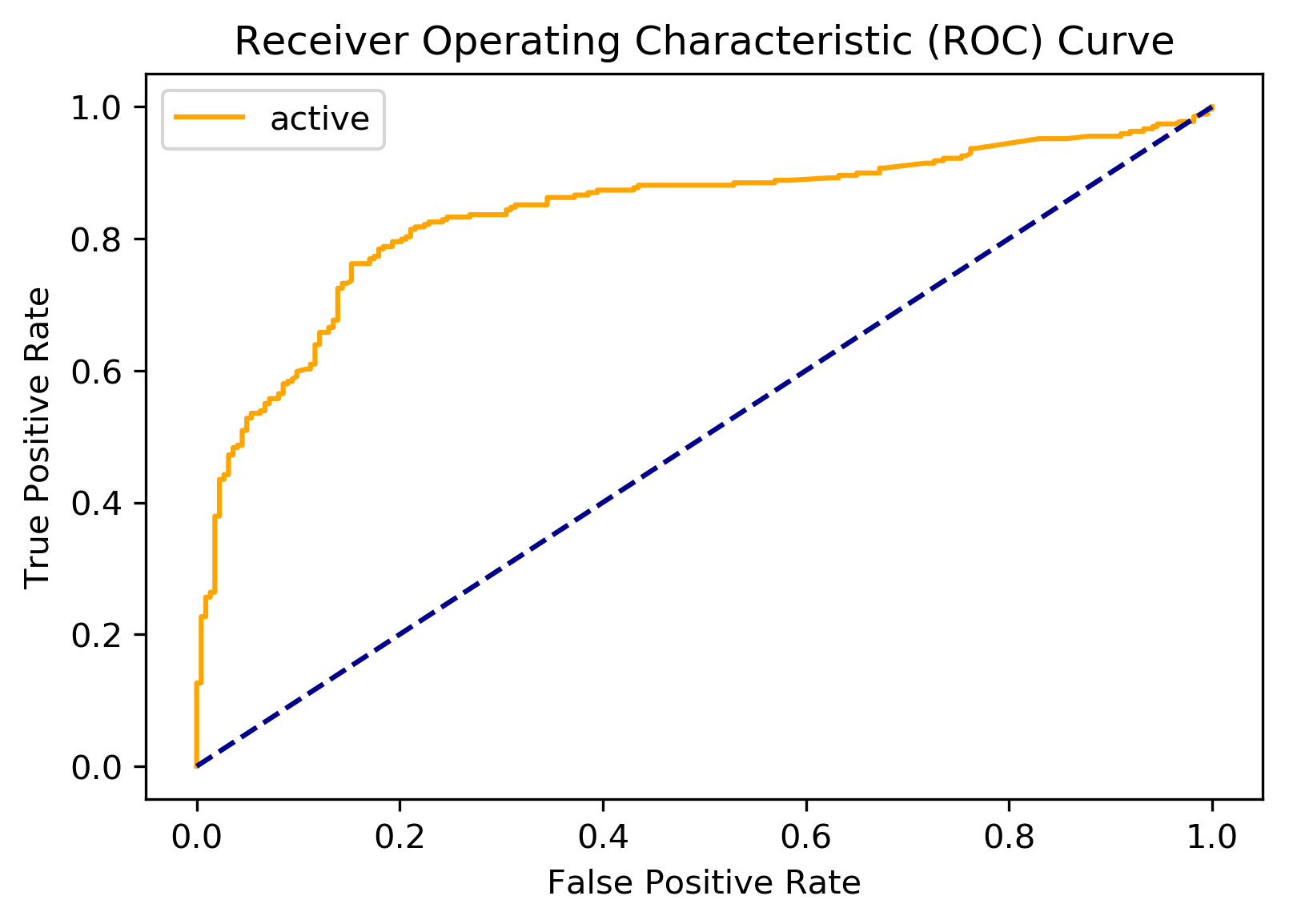


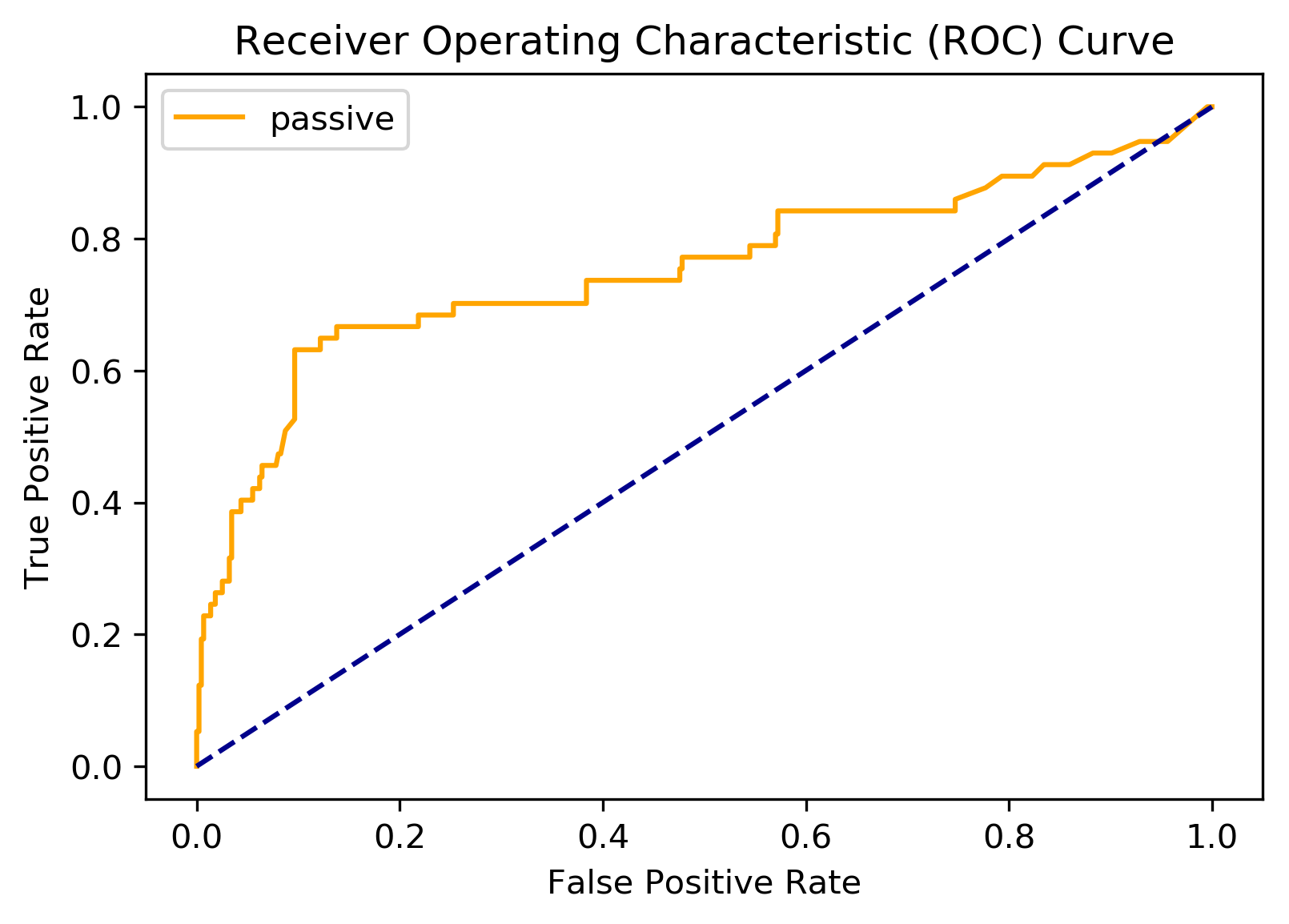


## Machine Learning Classifier Versus Manual Lexicon Approach Comparison

We compared the use of a manually created lexicon to that of our machine learning classifier. Specifically, while the full set of essays was in the process of being transcribed, two authors (JD and BP) manually created a lexicon (i.e., a list of words) indicative of physical activity, based on a subset of 179 essays available from a previous project (i.e., the Social Participation Study)^[[3]](#footnote-3)^. The final list of words attempted to consider risk of false positives and to identify words that could distinguish active and spectator identities. This resulted in a manually derived lexicon indicating physical activity identity (PAI).

| aerobic | archer | athleits |
| --- | --- | --- |
| athletics | badmi* | badminton |
| base ball | baseball | bicycl* |
| boxer | boxing | caving |
| climbing | cricket | criket |
| croket | croqet | croquet |
| cycl* | danc* | dance |
| dancing | diving | do sport |
| equestrian | exercis* | fell walking |
| football | golf | gym |
| hik* | hockey | hokey |
| horse | horse rid* | jog |
| net ball | netball | olympic |
| physical activity \| | rounders | rowing |
| rugby | running | sail* |
| scrambling | shooting | show jump |
| ski* | skin diving | soccer |
| squash | surf | swam |
| swim* | tennis | track and field |
| volleyball | walking |  |
| * indicates a wild card, which matches any suffix | | |

We then used the 500 labelled essays used in the “training set” to compare the manual lexicon with the machine learning approach described in the paper. Following the typical word count approach (used with the Linguistic Inquiry and Word Count program^[[4]](#footnote-4)^), we counted the relative frequency that the lexicon words appeared in the essays, compared to the number of words, and used the 500 activity labelled essays to determine which frequencies successfully distinguished between activity mentions or not. Using this ideal split point, we achieved accuracies of 54% for active (versus 75% for the machine learning model) and 80% spectator (versus 87.2% for the machine learning model), indicating that the machine learning approach more accurately captured active and spectator activities than the manually-derived lexicon. It is possible that the manual lexicon could be further refined to improve its accuracy, but such refinements require considerable time and resources, which are hard to justify compared to the speed and accuracy of the machine learning approach.

# S4. Physical Activity Trajectory Estimation Selection Process

PA trajectories were estimated using the age 33-55 dichotomized variables through latent class analysis, separately by gender. 2-class, 3-class, 4-class, 5-class and 6-class models were run and compared based on indicators of goodness of fit and interpretation of classes. As seen in Table S1, goodness of fit was similar across models. Entropy was unsatisfactory for all model specifications, hence we focused on the interpretation and meaning of trajectories.

We selected the four-class model. The 6-class model identified five main patterns, suggesting it was uninformative to go beyond five classes. On the other hand, the 2-class model failed to capture much of the variation occurring over time and appeared to hide relevant information. The 4-class model showed greater consistency across different samples (i.e., excluding those who migrated and died over the observation period or including them when using FIML. These results are available upon request). For completeness, Figure S2 illustrates physical activity trajectories for the 2, 3, 5, and 6 class models.

## Latent class model goodness of fit indicators by gender

| **Males** | | | |  | **Females** | | | | |
| --- | --- | --- | --- | --- | --- | --- | --- | --- | --- |
| **# classes** | **BIC** | **Entropy** | **Free parameters** | **Log-likelihood** | **# classes** | **BIC** | **Entropy** | **Free parameters** | **Log-likelihood** |
| 2 | 15422.98 | 0.503 | 9 | -7674.08 | 2 | 16063.82 | 0.482 | 9 | -7994.50 |
| 3 | 15411.42 | 0.678 | 14 | -7647.51 | 3 | 16071.07 | 0.539 | 14 | -7977.34 |
| 4 | 15445.07 | 0.505 | 19 | -7643.55 | 4 | 16103.17 | 0.466 | 19 | -7972.60 |
| 5 | 15486.63 | 0.593 | 24 | -7643.55 | 5 | 16144.73 | 0.426 | 24 | -7972.60 |
| 6 | 15528.20 | 0.568 | 29 | -7643.55 | 6 | 16186.30 | 0.493 | 29 | -7972.60 |

## Trajectories of physical activity from age 33 through age 55, based on 2, 3, 5 and 6-class models

| 2-class model |
| --- |
|  |

*Note.* See main paper for the selected 4-class model

| 3-class model |
| --- |
|  |

| 5-class model |
| --- |
|  |

| 6-class model |
| --- |
|  |

# S5. Latent Class Growth Analysis

Our analyses utilized longitudinal latent class analysis (LLCA), which requires fewer assumptions than generalized linear models such as growth mixed models and latent class growth analysis (LCGA). In addition, we conducted a sensitivity analysis in which we estimated trajectories of physical activity using LCGA.

As in LLCA, Latent Class Growth Analysis for categorical outcomes considers multiple *u* variables, seen as indicators of *c* and assumed conditionally independent given *c* (Muthen, 2004)^[[5]](#footnote-5)^. In LLCA, change over time can be modelled in piecewise fashion; the probability of being active at each age is estimated for each class through unrestricted, class-specific conditional probabilities. In LCGA, change is modelled parametrically; polynomial restrictions are placed on the shape of the outcome trajectories. Only LCGA takes advantage of time-ordered outcomes (Croudace et al., 2003).^[[6]](#footnote-6)^

As part of the sensitivity analysis, we tried to include covariates in the model, to reproduce the simultaneous estimation of classes and multinomial logistic regression. It is recommended that class enumeration is done prior to the inclusion of covariates (Nylund-Gibson & Masyn, 2016)^[[7]](#footnote-7)^, hence we included covariates in the four-class model, as we did when we used LLCA. However, results were highly unstable and in some cases, convergence was not achieved. It has been suggested that substantial changes in parameter values after covariates are included (e.g., omitting direct effects of covariates on observed variables) indicate misspecification of their effects (Feldman, Masyn & Conger, 2009)^[[8]](#footnote-8)^. Hence, we retained LLCA as the main method within the paper and we present here the estimation of classes using LCGA, without including covariates.

## Latent class model goodness of fit indicators by gender

|  | **# classes** | **BIC** | **entropy** | **Free parameters** | **Log-likelihood** |
| --- | --- | --- | --- | --- | --- |
| Males | 4 | 15444.08 | 0.675 | 11 | -7676.31 |
| Females | 4 | 16067.29 | 0.590 | 11 | -7987.92 |

## Trajectories of physical activity based on one-step model estimation, age 33-55, 4-class models

# S6. Simultaneous estimation of LCA and multinomial logistic regression model

As a sensitivity analysis, we estimated PA trajectories simultaneously in a single step, together with the multinomial model including all covariates. This was done separately by gender and for both specifications of the model: one considering active PAI as main predictor of adult PA trajectories, and the other including passive PAI. Below, we report goodness of fit indicators and graphs of trajectories.

Recurrent neural network techniques such as long short-term memory networks could provide a means to directly predict the observed PA reports at all the time-points, but interpretation of the effect of inputs to such models, especially in the context of control variables, is not clear. Future work may focus on producing the most accurate prediction of future PA rather than studying the relationship of specific predictors and utilize such powerful predictive techniques.

## Latent class model goodness of fit indicators by gender

|  |  | **# classes** | **BIC** | **entropy** | **Free parameters** |
| --- | --- | --- | --- | --- | --- |
| Males | active PAI included | 4 | 19670.7 | 0.829 | 54 |
|  | passive PAI included | 4 | 19528.77 | 0.685 | 54 |
| Females | active PAI included | 4 | 19762.79 | 0.647 | 54 |
|  | passive PAI included | 4 | 19703.86 | 0.751 | 53 |

## Trajectories of physical activity based on one-step model estimation, age 33-55, 4-class models

## Relative Risk Ratios (RRR) for the fully-adjusted model of PA trajectory classes compared to baseline trajectory class “always active”, separately by gender, one step.

| **VARIABLES** | **Male** | | | **Females** | | |
| --- | --- | --- | --- | --- | --- | --- |
|  | **Fluctuating/ increasing PA** | **Declining PA** | **Always inactive** | **Fluctuating/ increasing PA** | **Declining PA** | **Always inactive** |
| Active PAI (ref. No) | 0.731* | 0.844* | 0.710** | 0.673** | 0.754** | 0.873 |
| 95% CI | (0.524 - 1.020) | (0.691 - 1.030) | (0.535 - 0.941) | (0.481 -0.941) | (0.586 -0.968) | (0.657 -1.16) |
| Spectator PAI (ref. No) | 0.753 | 1.279* | 0.719 | 0.82 | 0 | 0.963 |
| 95% CI | (0.416 - 1.363) | (0.989 - 1.653) | (0.466 - 1.109) | (0.414 -1.624) | (0 -0) | (0.628 -1.476) |

*Note*. Reference group is no mention of active or spectator activities in the childhood essays. PA = physical activity; PAI=physical activity identity. 95% CI in brackets ()

^a^ Unreliable estimate, due to small class size

*** p<0.01, ** p<0.05, * p<0.1

# S7. Analytic Codes

## Stata Analysis Code

************************************************************************

* P H Y S I C A L A C T I V I T Y *

************************************************************************

* ------------------------------------------- Age 23 -------------------------------------------- *

use "ncds4.dta", clear

recode n5916 (1/3=1 "more than once a week") (4/6=0 "less than once a week"), gen (sport4)

keep ncdsid n5916 n622_4 n5739 sport4

* -------------------------------------------- Age 33 ------------------------------------------------ *

merge 1:1 ncdsid using "ncds5cmi.dta", keepusing (n504363 n504362 n622_5 n503913)

gen sport5=n504363

replace sport5=0 if n504362==2 & sport5==.

recode sport5 (1 2 3 4=1) (5 6=0)

label define sport 0 "less than once a week" 1 "more than once a week"

label value sport5 sport

label variable sport5 "How often undertakes sport/exercise/etc"

* ------------------------------- Age 42 ---------------------------------------------------- *

drop _merge

merge 1:1 ncdsid using "ncds6.dta", keepusing(breathls exercise n622_6 hlthgen )

gen sport6=breathls if breathls<7

replace sport6=0 if exercise==2 & sport6==.

recode sport6 (1 2 3 4=1) (5 6=0) (9=.)

label value sport6 sport

label variable sport6 "How often takes part in any exercise activity"

* ------------------------------ Age 46 ------------------------------ *

drop _merge

merge 1:1 ncdsid using "ncds7.dta", keepusing(n7exers1 n7breals nd7sex)

gen sport7=n7breals if n7breals>0

replace sport7=0 if n7exers1==2 & sport7==.

recode sport7 (1 2 3 4=1) (5 6=0)

label value sport7 sport

label variable sport7 "Frequency of exercising"

* ------------------------------ Age 50 ------------------------------ *

drop _merge

rename ncdsid NCDSID

merge 1:1 NCDSID using "ncds_2008_followup.dta" , keepusing(N8EXERSE N8BREALS N8SCQ1A ND8SEX N8HLTHGN)

renvars, lower

gen sport8=n8breals if n8breals>0

replace sport8=0 if n8exerse==2 & sport8==.

recode sport8 (1 2 3 4=1) (5 6=0)

label value sport8 sport

label variable sport8 "Frequency of exercising"

* ------------------------------ Age 55 ------------------------------ *

drop _merge

rename ncdsid NCDSID

merge 1:1 NCDSID using "ncds_2013_flatfile.dta", keepusing(N9LEIS01 N9CMSEX N9HLTHGN)

gen sport9=N9LEIS01 if N9LEIS01>0

recode sport9 (1=1) (2 3 4=0)

label value sport9 sport

label variable sport9 "Frequency of leisure activity - Play Sport Or Go Walking Or Swimming"

drop _merge

merge 1:1 NCDSID using "ncds_response.dta"

drop _merge

*drop if OUTCME09==8 OUTCME09==7

** Adding PA identity scores

rename NCDSID ncdsid

merge 1:1 ncdsid using "ncds0123.dta"

drop _merge

merge 1:1 ncdsid using "NCDS_PAI.dta" // this is the file with the PAI score (calculated for the research not available from UK Data Service)

** CONFOUNDERS

gen sport11= n941 if n941>0

label define sport11 1 "Most days" 2 "Sometimes" 3 "Hardly ever"

label value sport11 sport11

gen socclass11= n1687 if n1687>0 & n1687<7

label value socclass11 N1687

replace socclass=1 if n190==2 & socclass==. & n1687==.

replace socclass=2 if n190==3 & socclass==.& n1687==.

replace socclass=3 if n190==4 & socclass==.& n1687==.

replace socclass=4 if n190==5 & socclass==.& n1687==.

replace socclass=5 if n190==6 & socclass==.& n1687==.

replace socclass=5 if n190==7 & socclass==.& n1687==.

replace socclass=6 if n190==8 & socclass==.& n1687==.

gen a= n236-1

replace socclass=a if socclass==. & a>0

* Missschool – recoded from n1301 as shown in the table below

clonevar miss_school11=n1301

replace miss_school11=. if n1301==-1 n1301==5 n1301==6 n1301==7

* Hospital – recoded from n1400 as shown in the table below

clonevar nhosp11=n1400

replace nhosp11=. if n1400==-1

*Self-rated health

gen srh4= n5739 if n5739<5

label value srh N5739

gen srh5=n503913

label value srh5 n503913

gen srh6=hlthgen if hlthgen<5

label value srh6 HLTHGEN

gen srh8=n8hlthgn if n8hlthgn>0

label value srh8 N8HLTHGN

gen srh9=N9HLTHGN if N9HLTHGN>0

label value srh9 N9HLTHGN

* BMI

gen weight_11=dvwt11

replace weight_11=. if dvwt11<0

gen height_11=dvht11

replace height_11=. if dvht11<0

gen bmi_11=weight_11/(height_11*height_11)

replace bmi_11=. if weight_11==.|height_11==.

*Birthweight

gen birthweight=n646

replace birthweight=. if n646<0

replace birthweight=88+int((200-88+1)*runiform()) if n646==508

replace birthweight=40+int((88-40+1)*runiform()) if n646==509

*Smoking during pregnancy

gen smoke_preg=.

replace smoke_preg=0 if n502==1 & n503==1

replace smoke_preg=0 if n503==-1

replace smoke_preg=0 if n503==2

replace smoke_preg=1 if n502!=1 & n503==1

replace smoke_preg=1 if n503==3|n503==4|n503==5|n503==6|n503==7|n503==8|n503==9|n503==10

replace smoke_preg=. if n503==.

*BSAG Syndromes scores

gen bsga_tot11=n1008

replace bsga_tot11=. if bsga_tot11<0

*Enuresis Age 11 (n1271 completely dry at night, n1272 completely dry during the day)

gen enuresis11=(n1271==2|n1271==3|n1271==4|n1271==5)

replace enuresis11=. if (n1271==-1|n1271==6|n1271==.)

replace enuresis11=1 if n1272==2

*RUTTER SCORE AGE 11

drop _merge

rename ncdsid NCDSID

merge 1:1 NCDSID using ""$path1\derivedvariables.dta" , keepusing(ZNCDS7PD ZNCDS11PD) // this datasets includes derived variables from previous research

gen rutterpd11 = ZNCDS11PD

replace rutterpd11=. if ZNCDS11PD==9999

*General ability

gen genability11=n920

replace genability11=. if genability11<0

*Physical coordination Age 11 (n882 Child has poor physical coordination)

gen phycoord11=(n882==1|n882==2)

replace phycoord11=. if n882==-1|n882==4|n882==.

/** -------------------------- SAMPLE --------------------------------------- *

Sample are those completing essays and having at least one info on PA in adulthood, who did not die or emigrate during the observation time point N=8,866 */

keep if active!=. passive!=.

keep if sport4!=. sport5!=. sport6!=. sport8!=. |sport9!=.

drop if OUTCME09==7 OUTCME09==8

encode NCDSID, gen(id)

** Convert to mplus

stata2mplus sport4 sport5 sport6 sport8 sport9 ///

active passive id N622 sport11 socclass11 srh4 srh5 srh6 srh8 srh9 bmi_11 birthweight smoke_preg ///

bsga_tot11 rutterpd11 genability11 enuresis11 phycoord11 n236 n90 ///

miss_school11 nhosp11 n455 n923 unemtime using "$path1 \mi_mplus", replace

*************************************

* VARIABLES TO BE USED for MI *

*************************************

--> Auxiliary variables */

global path2 "C:\Users\CLS\Cohort studies\NCDS"

merge 1:1 ncdsid using "$casa\ncds0123.dta", keepusing (n646 n639 n236 n90 n920 n455 n923)

drop if _merge==2

drop _merge

merge 1:1 ncdsid using "$casa\ncds4.dta" , keepusing (n5916 unemtime)

drop if _merge==2

drop _merge

* clean variables:

recode n646 (-1=.) // not needed=bweight

recode n639 (-1=.) // not needed=smoke_pre

recode n236 (-1=.) (1=8)

recode n90 (-1=.)

recode n920 (-1=.) // not needed=genability

recode n455 (-1=.)

recode n923 (-1=.)

recode unemtime (-1=.)

merge 1:1 ncdsid using "$casa\PA_4class" // add PA latent class to the dataset

** MULTIPLE IMPUTATION **

set matsize 1000

mi set wide

mi register imputed sport4 sport5 sport6 sport8 sport9 active passive

sport11 socclass11 srh4 srh5 srh6 srh8 srh9 bmi_11 birthweight smoke_preg ///

bsga_tot11 rutterpd11 genability11 enuresis11 phycoord11 n236 n90 ///

miss_school11 nhosp11 n455 n923 unemtime

mi set M=50

mi impute chained (regress) bmi_11 birthweight bsga_tot11 rutterpd11 genability11 ///

n455 n923 unemtime n90 n236 miss_school11 nhosp11 ///

(ologit) sport11 socclass11 srh4 srh5 srh6 srh8 srh9 (mlogit) ///

(logit) sport4 sport5 sport6 sport8 sport9 active passive smoke_preg enuresis11 ///

phycoord11, replace noisily augment

save mi_8866sample.dta, replace

*To export multiple imputed datasets in mplus (For Sensitivity Analysis)

mi export nhanes1 mistata8158

******************************

**** REGRESSIONS ***

******************************

* model 1: full model in the paper

* Males

forvalues i=4/6{

eststo: mi estimate, esampvaryok post: logit sport`i' i.active i.sport11 i.socclass11 ///

bmi_11 birthweight smoke_preg miss_school11 nhosp11 ///

bsga_tot11 rutterpd11 genability11 enuresis11 phycoord11 if N622==1, or

outreg2 using fullmodel, ci eform

}

forvalues i=8/9{

eststo: mi estimate, esampvaryok post: logit sport`i' i.active i.sport11 i.socclass11 ///

bmi_11 birthweight smoke_preg miss_school11 nhosp11 ///

bsga_tot11 rutterpd11 genability11 enuresis11 phycoord11 if N622==1, or

outreg2 using fullmodel, ci eform

}

forvalues i=4/6{

eststo: mi estimate, esampvaryok post: logit sport`i' i.passive i.sport11 i.socclass11 ///

bmi_11 birthweight smoke_preg miss_school11 nhosp11 ///

bsga_tot11 rutterpd11 genability11 enuresis11 phycoord11 if N622==1, or

outreg2 using fullmodel, ci eform

}

forvalues i=8/9{

eststo: mi estimate, esampvaryok post: logit sport`i' i.passive i.sport11 i.socclass11 ///

bmi_11 birthweight smoke_preg miss_school11 nhosp11 ///

bsga_tot11 rutterpd11 genability11 enuresis11 phycoord11 if N622==1, or

outreg2 using fullmodel, ci eform

}

* Females

forvalues i=4/6{

eststo: mi estimate, esampvaryok post: logit sport`i' i.active i.sport11 i.socclass11 ///

bmi_11 birthweight smoke_preg miss_school11 nhosp11 ///

bsga_tot11 rutterpd11 genability11 enuresis11 phycoord11 if N622==2, or

outreg2 using fullmodel, ci eform

}

forvalues i=8/9{

eststo: mi estimate, esampvaryok post: logit sport`i' i.active i.sport11 i.socclass11 ///

bmi_11 birthweight smoke_preg miss_school11 nhosp11 ///

bsga_tot11 rutterpd11 genability11 enuresis11 phycoord11 if N622==2, or

outreg2 using fullmodel, ci eform

}

forvalues i=4/6{

eststo: mi estimate, esampvaryok post: logit sport`i' i.passive i.sport11 i.socclass11 ///

bmi_11 birthweight smoke_preg miss_school11 nhosp11 ///

bsga_tot11 rutterpd11 genability11 enuresis11 phycoord11 if N622==2, or

outreg2 using fullmodel, ci eform

}

forvalues i=8/9{

eststo: mi estimate, esampvaryok post: logit sport`i' i.passive i.sport11 i.socclass11 ///

bmi_11 birthweight smoke_preg miss_school11 nhosp11 ///

bsga_tot11 rutterpd11 genability11 enuresis11 phycoord11 if N622==2, or

outreg2 using fullmodel, ci eform

}

** Model 2 no confounders

* Males

forvalues i=4/6{

eststo: mi estimate, esampvaryok post: logit sport`i' i.active if N622==1, or

outreg2 using noconf, ci eform

}

forvalues i=8/9{

eststo: mi estimate, esampvaryok post: logit sport`i' i.active if N622==1, or

outreg2 using noconf, ci eform

}

forvalues i=4/6{

eststo: mi estimate, esampvaryok post: logit sport`i' i.passive if N622==1, or

outreg2 using noconf, ci eform

}

forvalues i=8/9{

eststo: mi estimate, esampvaryok post: logit sport`i' i.passive if N622==1, or

outreg2 using noconf, ci eform

}

* Females

forvalues i=4/6{

eststo: mi estimate, esampvaryok post: logit sport`i' i.active if N622==2, or

outreg2 using noconf, ci eform

}

forvalues i=8/9{

eststo: mi estimate, esampvaryok post: logit sport`i' i.active if N622==2, or

outreg2 using noconf, ci eform

}

forvalues i=4/6{

eststo: mi estimate, esampvaryok post: logit sport`i' i.passive if N622==2, or

outreg2 using noconf, ci eform

}

forvalues i=8/9{

eststo: mi estimate, esampvaryok post: logit sport`i' i.passive if N622==2, or

outreg2 using noconf, ci eform

}

***** Model 3 no sport age 11

* Males

forvalues i=4/6{

eststo: mi estimate, esampvaryok post: logit sport`i' i.active i.socclass11 ///

bmi_11 birthweight smoke_preg miss_school11 nhosp11 ///

bsga_tot11 rutterpd11 genability11 enuresis11 phycoord11 if N622==1, or

outreg2 using nosport11, ci eform

}

forvalues i=8/9{

eststo: mi estimate, esampvaryok post: logit sport`i' i.active i.socclass11 ///

bmi_11 birthweight smoke_preg miss_school11 nhosp11 ///

bsga_tot11 rutterpd11 genability11 enuresis11 phycoord11 if N622==1, or

outreg2 using nosport11, ci eform

}

forvalues i=4/6{

eststo: mi estimate, esampvaryok post: logit sport`i' i.passive i.socclass11 ///

bmi_11 birthweight smoke_preg miss_school11 nhosp11 ///

bsga_tot11 rutterpd11 genability11 enuresis11 phycoord11 if N622==1, or

outreg2 using nosport11, ci eform

}

forvalues i=8/9{

eststo: mi estimate, esampvaryok post: logit sport`i' i.passive i.socclass11 ///

bmi_11 birthweight smoke_preg miss_school11 nhosp11 ///

bsga_tot11 rutterpd11 genability11 enuresis11 phycoord11 if N622==1, or

outreg2 using nosport11, ci eform

}

* Females

forvalues i=4/6{

eststo: mi estimate, esampvaryok post: logit sport`i' i.active i.socclass11 ///

bmi_11 birthweight smoke_preg miss_school11 nhosp11 ///

bsga_tot11 rutterpd11 genability11 enuresis11 phycoord11 if N622==2, or

outreg2 using nosport11, ci eform

}

forvalues i=8/9{

eststo: mi estimate, esampvaryok post: logit sport`i' i.active i.socclass11 ///

bmi_11 birthweight smoke_preg miss_school11 nhosp11 ///

bsga_tot11 rutterpd11 genability11 enuresis11 phycoord11 if N622==2, or

outreg2 using nosport11, ci eform

}

forvalues i=4/6{

eststo: mi estimate, esampvaryok post: logit sport`i' i.passive i.socclass11 ///

bmi_11 birthweight smoke_preg miss_school11 nhosp11 ///

bsga_tot11 rutterpd11 genability11 enuresis11 phycoord11 if N622==2, or

outreg2 using nosport11, ci eform

}

forvalues i=8/9{

eststo: mi estimate, esampvaryok post: logit sport`i' i.passive i.socclass11 ///

bmi_11 birthweight smoke_preg miss_school11 nhosp11 ///

bsga_tot11 rutterpd11 genability11 enuresis11 phycoord11 if N622==2, or

outreg2 using nosport11, ci eform

}

*** Trajectories

* To estimate the trajectories, I used the classes created in Mplus using FIML

* merge 1:1 ncdsid using...

eststo: mi estimate, esampvaryok post: mlogit Cnew i.active i.sport11 i.socclass11 ///

bmi_11 birthweight smoke_preg miss_school11 nhosp11 ///

bsga_tot11 rutterpd11 genability11 enuresis11 phycoord11 if N622==1

outreg2 using trajectoryPA, ci eform

eststo: mi estimate, esampvaryok post: mlogit Cnew i.active i.sport11 i.socclass11 ///

bmi_11 birthweight smoke_preg miss_school11 nhosp11 ///

bsga_tot11 rutterpd11 genability11 enuresis11 phycoord11 if N622==2

outreg2 using trajectoryPA, ci eform

eststo: mi estimate, esampvaryok post: mlogit Cnew i.passive i.sport11 i.socclass11 ///

bmi_11 birthweight smoke_preg miss_school11 nhosp11 ///

bsga_tot11 rutterpd11 genability11 enuresis11 phycoord11 if N622==1

outreg2 using trajectoryPA, ci eform

eststo: mi estimate, esampvaryok post: mlogit Cnew i.passive i.sport11 i.socclass11 ///

bmi_11 birthweight smoke_preg miss_school11 nhosp11 ///

bsga_tot11 rutterpd11 genability11 enuresis11 phycoord11 if N622==2

outreg2 using trajectoryPA, ci eform

## Mplus Analysis Code

*Latent class analysis for physical activity patterns. Codes reported for females.*

Data:

File is PA.dat;

Variable:

Names are

sport5 sport6 sport7 sport8 sport9 N622 ncdsid;

Missing are all (-9999) ;

Usevariables are sport5 sport6 sport8 sport9;

Categorical are sport5 sport6 sport8 sport9;

Classes=c(4);

useobservation are (N622 eq 2);

idvariable is NCDSID;

Analysis:

Type = mixture;

starts = 500 70;

Model:

%overall%

Plot:

type is plot2 plot3;

Series is sport5-sport9 (*);

Output:

sampstat tech8 tech10 tech14 patterns;

SAVEDATA:

file is paC4_f.txt ;

save is cprob;

format is free;

*Latent class growth analysis for physical activity patterns. Codes reported for females.*

File is PA.dat ;

Variable:

Names are

sport5 sport6 sport7 sport8 sport9 N622 id;

Missing are all (-9999) ;

Usevariables are sport5 sport6 sport8 sport9 ;

Categorical= sport5 sport6 sport8 sport9 ;

Classes=c(4);

useobservation are (N622 eq 2);

idvariable is id;

Analysis:

Type=mixture ;

starts= 500 70;

Model:

%overall%

i s | sport5@0 sport6@1 sport8@2 sport9@3;

Plot:

type is plot2 plot3;

Series is sport5-sport9 (s);

Output:

sampstat tech8 tech10 SVALUES;

SAVEDATA:

file is lcga_f4c_8158.txt ;

save is cprob;

format is free;

*Simultaneous estimation of LCA and multinomial logistic regression model. Code reported for females and active PAI. The other cases were based on the same syntax.*

Data:

FILE IS mistatalist.dat; ! multiple imputed dataset produced in Stata

TYPE = IMPUTATION;

Variable:

Names are

sport4mi sport5mi sport6mi sport8mi sport9mi N622

n236mi n455mi n90mi n923mi activemi passivemi

sport11mi socclass11mi miss_school11mi nhosp11mi

srh4mi srh5mi srh6mi srh8mi srh9mi bmi_11mi

birthweightmi smoke_pregmi bsga_tot11mi enuresis11mi

rutterpd11mi genability11mi phycoord11mi

Cmi unemtimemi id;

Missing are all (-9999) ;

Usevariables are sport5mi sport6mi sport8mi sport9mi activemi

sport11mi miss_school11mi nhosp11mi bmi_11mi birthweightmi

smoke_pregmi bsga_tot11mi enuresis11mi rutterpd11mi

genability11mi phycoord11mi ses1 ses2 ses3 ses4 ses5 ;

Categorical are sport5mi sport6mi sport8mi sport9mi;

Classes=c(4);

useobservation are (N622 eq 2);

idvariable is id;

define :

ses1=socclass11mi==1;

ses2=socclass11mi==2;

ses3=socclass11mi==3;

ses4=socclass11mi==4;

ses5=socclass11mi==5;

Analysis:

Type = mixture;

ESTIMATOR = ML;

Model:

%overall%

c#1 on activemi (a)

sport11mi ses1 ses2 ses3 ses4 ses5

bmi_11mi birthweightmi smoke_pregmi miss_school11mi nhosp11mi

bsga_tot11mi rutterpd11mi genability11mi enuresis11mi phycoord11mi;

c#2 on activemi (b)

sport11mi ses1 ses2 ses3 ses4 ses5

bmi_11mi birthweightmi smoke_pregmi miss_school11mi nhosp11mi

bsga_tot11mi rutterpd11mi genability11mi enuresis11mi phycoord11mi;

c#3 on activemi (c)

sport11mi ses1 ses2 ses3 ses4 ses5

bmi_11mi birthweightmi smoke_pregmi miss_school11mi nhosp11mi

bsga_tot11mi rutterpd11mi genability11mi enuresis11mi phycoord11mi;

! we fix starting values to have “always active” as reference group

%c#4%

[SPORT5MI$1@-1.92];

[SPORT6MI$1@-2.078];

[SPORT8MI$1@-7.766];

[SPORT9MI$1@-15];

%c#2%

[SPORT5MI$1@-1.132];

[SPORT6MI$1@-1.034];

[SPORT8MI$1@-0.427];

[SPORT9MI$1@1.59];

%c#3%

[SPORT5MI$1@0.285];

[SPORT6MI$1@0.395];

[SPORT8MI$1@-0.497];

[SPORT9MI$1@-15];

%c#1%

[SPORT5MI$1@0.655];

[SPORT6MI$1@1.323];

[SPORT8MI$1@1.145];

[SPORT9MI$1@15];

MODEL CONSTRAINT:

NEW(orDa orDb orDc);

orDa=exp(a);

orDb=exp(b);

orDc=exp(c);

Plot:

type is plot2 plot3;

Series is sport5mi-sport9mi (*);

Output:

sampstat tech14 tech8 tech10;

SAVEDATA:

file is 1step_MI_f_active.txt ;

save is cprob;

format is free;

## Differential Language Analysis ToolKit (DLATK) Code

**List of commands for the language analysis and machine learning.**

*All analyses below were performed using the python package, “Differential Language Analysis ToolKit (DLATK). The package is openly available here:* [*http://dlatk.wwbp.org/*](http://dlatk.wwbp.org/) *. It has been used in dozens of peer-reviewed publications across psychology, computer science, and behavioral health domains (see* [*http://dlatk.wwbp.org/papers.html*](http://dlatk.wwbp.org/papers.html)*).*

**PART I. Linguistic Feature Extraction**

**1. Generate variables encoding whether each unigram (single words, also known as 1grams) existed or not in each essay.**

*./dlatkInterface.py -d ncds -t s2_essays_v3 -c user_id --add_ngrams -n 1 2 3 --boolean --combine_feats 1to3gram*

**(**produces: feat$1to3gram$s2_essays_v3$user_id$16to1 which contains all unigrams**)**

**2. Filter to unigrams mentioned in at least 0.1% (1 out of every 1000) essays:**

*./dlatkInterface.py -d ncds -t s2_essays_v3 -c user_id --group_freq_thresh 50 --feat_occ_filter --set_p_occ .001 -f ‘feat$1gram$s2_essays_v3$user_id$16to1’*

**(**produces: feat$1to3gram$s2_essays_v3$user_id$16to1 which contains the subset of unigrams; group frequency threshold limits to essays with at least 50 words**)**

**PART II. Machine Learning**

**1. Cross-validating the model (linear SVM) using 10-folds, where regularization parameters are set on training only:**

*./dlatkInterface.py -d ncds -t s2_essays_v3 -c user_id -f 'feat$1to3gram$s2_essays_v3$user_id$16to1$0_05' --outcome_table physical_activity_ratings --outcomes active passive act_and_pass act_or_pass act_not_pass --combo_test_classifier --model linear-svc --group_freq_thresh 50 --folds 10*

**2. Save the model to a “pickle file”.**

*./dlatkInterface.py -d ncds -t s2_essays_v3 -c user_id -f 'feat$1to3gram$s2_essays_v3$user_id$16to1$0_05' --outcome_table physical_activity_ratings --outcomes active passive act_and_pass act_or_pass act_not_pass --train_classifier --model linear-svc --group_freq_thresh 50 --save_model --picklefile [DIRECTORY]/physical_activity_ratings.1to3grams_boolean.linear_svc.group50.pickle*

**3. Loading the pickle file and running it across *all* of the s2_essays_v3 table to generate annotations for all the users.**

*./dlatkInterface.py -d ncds -t s2_essays_v3 -c user_id -f 'feat$1to3gram$s2_essays_v3$user_id$16to1$0_05' --group_freq_thresh 50 --load_model --picklefile [DIRECTORY]/physical_activity_ratings.1to3grams_boolean.linear_svc.group50.pickle*

*--predict_classifiers_to_outcome_table activity*

The mysql table “activity” now contains the predictions for active and passive activity for each essay. This can then be exported as a csv file and analyzed in any statistical software.

**ROC curve estimation**

*./dlatkInterface.py -d ncds -t s2_essays_v3 -c user_id -f 'feat$1to3gram$s2_essays_v3$user_id$16to1$0_02' 'feat$meta_1gram$s2_essays_v3$user_id$16to16' --outcome_table physical_activity_ratings --outcomes active passive --nfold_classif --model linear-svc --group_freq_thresh 50 --folds 10 --stratify_folds --prob_csv --pred_csv --output_name /data/ncds/results/pa_classifier_probs_19-12-2*

# S8. Odd ratios predicting adult activity from childhood active and passive PAI

## Odds ratios predicting adult activity from childhood active PAI, males.

| **VARIABLES** | **PA age 23** | **PA age 33** | **PA age 42** | **PA age 50** | **PA age 55** |
| --- | --- | --- | --- | --- | --- |
| Active PAI (ref. No) | 1.378*** | 1.135* | 1.252*** | 1.210** | 1.194** |
|  | (1.202 - 1.580) | (0.977 - 1.319) | (1.078 - 1.455) | (1.039 - 1.409) | (1.020 - 1.397) |
| Sport out of school age 11 - sometimes (Ref most days) | 0.676*** | 0.852** | 0.887 | 0.918 | 0.815** |
|  | (0.584 - 0.781) | (0.726 - 0.999) | (0.761 - 1.034) | (0.776 - 1.088) | (0.695 - 0.956) |
| Sport out of school age 11 - hardly ever (Ref most days) | 0.462*** | 0.686*** | 0.704*** | 0.774* | 0.817 |
|  | (0.354 - 0.603) | (0.526 - 0.895) | (0.549 - 0.904) | (0.593 - 1.010) | (0.626 - 1.066) |
| Father's social class in childhood (ref. I): II | 0.848 | 0.982 | 1.083 | 0.925 | 0.882 |
|  | (0.626 - 1.148) | (0.708 - 1.361) | (0.774 - 1.515) | (0.633 - 1.354) | (0.619 - 1.256) |
| Father's social class in childhood: III non manual | 0.85 | 1.015 | 1.047 | 0.817 | 0.711* |
|  | (0.608 - 1.187) | (0.704 - 1.464) | (0.730 - 1.501) | (0.547 - 1.218) | (0.487 - 1.037) |
| Father's social class in childhood: III manual | 0.865 | 0.993 | 0.835 | 0.695** | 0.607*** |
|  | (0.651 - 1.149) | (0.733 - 1.345) | (0.612 - 1.140) | (0.492 - 0.983) | (0.433 - 0.852) |
| Father's social class in childhood: IV | 0.775 | 1.021 | 0.947 | 0.753 | 0.549*** |
|  | (0.565 - 1.062) | (0.725 - 1.438) | (0.674 - 1.330) | (0.514 - 1.103) | (0.386 - 0.782) |
| Father's social class in childhood: V | 0.948 | 0.914 | 0.761 | 0.659* | 0.585** |
|  | (0.645 - 1.395) | (0.602 - 1.388) | (0.499 - 1.160) | (0.422 - 1.029) | (0.367 - 0.931) |
| BMI at age 11 | 0.985 | 1.008 | 0.997 | 0.99 | 0.991 |
|  | (0.956 - 1.014) | (0.978 - 1.040) | (0.965 - 1.030) | (0.958 - 1.024) | (0.961 - 1.022) |
| Birth weight | 1 | 0.998 | 1 | 1 | 1.001 |
|  | (0.997 - 1.004) | (0.994 - 1.002) | (0.996 - 1.003) | (0.996 - 1.004) | (0.997 - 1.005) |
| Smoke during pregnancy (ref. No) | 1.11 | 0.962 | 1.004 | 1.054 | 0.925 |
|  | (0.959 - 1.286) | (0.820 - 1.128) | (0.857 - 1.176) | (0.890 - 1.249) | (0.784 - 1.091) |
| Time off school for ill health in the past year at age 11 | 0.965 | 0.857** | 0.869** | 0.872** | 0.895* |
|  | (0.858 - 1.085) | (0.759 - 0.969) | (0.771 - 0.981) | (0.771 - 0.987) | (0.792 - 1.011) |
| # of times child admitted to hospital at age 11 | 0.992 | 1.003 | 1.02 | 1.046 | 1.022 |
|  | (0.928 - 1.060) | (0.932 - 1.080) | (0.951 - 1.094) | (0.970 - 1.127) | (0.954 - 1.094) |
| BSAG score at age 11 | 0.990** | 0.990** | 0.989** | 0.996 | 0.994 |
|  | (0.982 - 0.998) | (0.981 - 0.999) | (0.981 - 0.998) | (0.987 - 1.005) | (0.984 - 1.004) |
| Rutter test at age 11 | 0.941* | 0.98 | 0.944 | 0.931* | 0.944 |
|  | (0.878 - 1.008) | (0.909 - 1.056) | (0.879 - 1.014) | (0.859 - 1.010) | (0.875 - 1.019) |
| General ability at age 11 | 1.007*** | 1.007** | 1.005** | 1.009*** | 1.005* |
|  | (1.002 - 1.012) | (1.001 - 1.012) | (1.000 - 1.011) | (1.003 - 1.014) | (0.999 - 1.010) |
| Enuresis at age 11 | 0.907 | 0.699** | 0.773* | 0.807 | 0.741** |
|  | (0.686 - 1.200) | (0.528 - 0.927) | (0.575 - 1.037) | (0.598 - 1.087) | (0.554 - 0.990) |
| Physical coordination (ref. Not poor) | 0.668*** | 0.829* | 0.959 | 0.843 | 0.738*** |
|  | (0.546 - 0.816) | (0.676 - 1.016) | (0.783 - 1.174) | (0.673 - 1.056) | (0.596 - 0.914) |
| N Observations | 4474 | 4474 | 4474 | 4474 | 4474 |

*Note*. PA = physical activity, PAI = physical activity identity, BSAG = Bristol Social Adjustment Guides, BMI = body mass index, Ref = reference group. Where no reference group is indicated, the variable is treated as continuous. 95% confidence intervals reported in brackets ().

* p < .05; ** p < .01, *** p < .001

## Odds ratios predicting adult activity from childhood passive PAI, males.

| **VARIABLES** | **PA age 23** | **PA age 33** | **PA age 42** | **PA age 50** | **PA age 55** |
| --- | --- | --- | --- | --- | --- |
| Spectator PAI (ref. No) | 1.331*** | 1.141 | 1.077 | 1.115 | 0.886 |
|  | (1.136 - 1.560) | (0.954 - 1.363) | (0.909 - 1.277) | (0.919 - 1.353) | (0.736 - 1.065) |
| Sport out of school age 11 - sometimes (Ref most days) | 0.666*** | 0.848** | 0.869* | 0.905 | 0.790*** |
|  | (0.576 - 0.770) | (0.723 - 0.995) | (0.746 - 1.011) | (0.765 - 1.072) | (0.674 - 0.925) |
| Sport out of school age 11 - hardly ever (Ref most days) | 0.451*** | 0.681*** | 0.681*** | 0.756** | 0.778* |
|  | (0.346 - 0.589) | (0.522 - 0.887) | (0.532 - 0.873) | (0.580 - 0.987) | (0.597 - 1.014) |
| Father's social class in childhood (ref. I): II | 0.843 | 0.978 | 1.084 | 0.924 | 0.89 |
|  | (0.623 - 1.142) | (0.705 - 1.356) | (0.775 - 1.515) | (0.631 - 1.351) | (0.625 - 1.268) |
| Father's social class in childhood: III non manual | 0.846 | 1.009 | 1.051 | 0.815 | 0.724* |
|  | (0.605 - 1.181) | (0.699 - 1.455) | (0.733 - 1.506) | (0.546 - 1.218) | (0.496 - 1.057) |
| Father's social class in childhood: III manual | 0.867 | 0.992 | 0.841 | 0.697** | 0.617*** |
|  | (0.653 - 1.152) | (0.732 - 1.344) | (0.616 - 1.148) | (0.493 - 0.986) | (0.440 - 0.865) |
| Father's social class in childhood: IV | 0.781 | 1.022 | 0.953 | 0.756 | 0.556*** |
|  | (0.570 - 1.070) | (0.725 - 1.440) | (0.678 - 1.339) | (0.516 - 1.107) | (0.391 - 0.790) |
| Father's social class in childhood: V | 0.958 | 0.915 | 0.77 | 0.664* | 0.596** |
|  | (0.651 - 1.409) | (0.603 - 1.390) | (0.506 - 1.173) | (0.426 - 1.036) | (0.374 - 0.949) |
| BMI at age 11 | 0.986 | 1.009 | 0.998 | 0.991 | 0.991 |
|  | (0.958 - 1.015) | (0.978 - 1.041) | (0.966 - 1.030) | (0.959 - 1.025) | (0.961 - 1.022) |
| Birth weight | 1 | 0.998 | 1 | 1 | 1.001 |
|  | (0.997 - 1.004) | (0.994 - 1.002) | (0.996 - 1.003) | (0.996 - 1.004) | (0.997 - 1.005) |
| Smoke during pregnancy (ref. No) | 1.106 | 0.96 | 1.002 | 1.052 | 0.925 |
|  | (0.956 - 1.281) | (0.819 - 1.126) | (0.856 - 1.174) | (0.888 - 1.247) | (0.784 - 1.091) |
| Time off school for ill health in the past year at age 11 | 0.962 | 0.856** | 0.866** | 0.870** | 0.891* |
|  | (0.856 - 1.081) | (0.758 - 0.967) | (0.768 - 0.977) | (0.769 - 0.984) | (0.789 - 1.006) |
| # of times child admitted to hospital at age 11 | 0.997 | 1.005 | 1.022 | 1.048 | 1.02 |
|  | (0.933 - 1.065) | (0.934 - 1.083) | (0.953 - 1.096) | (0.972 - 1.129) | (0.953 - 1.093) |
| BSAG score at age 11 | 0.990** | 0.990** | 0.989** | 0.996 | 0.993 |
|  | (0.982 - 0.998) | (0.981 - 0.999) | (0.980 - 0.998) | (0.987 - 1.005) | (0.984 - 1.003) |
| Rutter test at age 11 | 0.939* | 0.979 | 0.943 | 0.931* | 0.945 |
|  | (0.877 - 1.006) | (0.909 - 1.055) | (0.878 - 1.013) | (0.858 - 1.009) | (0.876 - 1.020) |
| General ability at age 11 | 1.007*** | 1.007** | 1.006** | 1.009*** | 1.005* |
|  | (1.003 - 1.012) | (1.002 - 1.012) | (1.001 - 1.011) | (1.004 - 1.015) | (1.000 - 1.011) |
| Enuresis at age 11 | 0.904 | 0.699** | 0.773* | 0.806 | 0.743** |
|  | (0.684 - 1.196) | (0.527 - 0.926) | (0.576 - 1.037) | (0.598 - 1.086) | (0.556 - 0.994) |
| Physical coordination (ref. Not poor) | 0.666*** | 0.829* | 0.949 | 0.839 | 0.724*** |
|  | (0.545 - 0.814) | (0.676 - 1.017) | (0.775 - 1.163) | (0.669 - 1.051) | (0.585 - 0.896) |
| N Observations | 4474 | 4474 | 4474 | 4474 | 4474 |

*Note*. PA = physical activity, PAI = physical activity identity, BSAG = Bristol Social Adjustment Guides, BMI = body mass index, Ref = reference group. Where no reference group is indicated, the variable is treated as continuous. 95% confidence intervals reported in brackets ().

* p < .05; ** p < .01, *** p < .001

## Odds ratios predicting adult activity from childhood active PAI, females.

| **VARIABLES** | **PA age 23** | **PA age 33** | **PA age 42** | **PA age 50** | **PA age 55** |
| --- | --- | --- | --- | --- | --- |
| Active PAI (ref. No) | 1.074 | 1.198** | 1.066 | 1.024 | 1.093 |
|  | (0.907 - 1.272) | (1.024 - 1.401) | (0.911 - 1.248) | (0.871 - 1.204) | (0.933 - 1.280) |
| Sport out of school age 11 - sometimes (Ref most days) | 0.779*** | 1.052 | 0.88 | 1.037 | 0.938 |
|  | (0.657 - 0.923) | (0.895 - 1.237) | (0.754 - 1.027) | (0.881 - 1.221) | (0.807 - 1.090) |
| Sport out of school age 11 - hardly ever (Ref most days) | 0.552*** | 0.826* | 0.983 | 0.973 | 0.982 |
|  | (0.412 - 0.741) | (0.659 - 1.037) | (0.783 - 1.234) | (0.771 - 1.227) | (0.784 - 1.230) |
| Father's social class in childhood (ref. I): II | 0.651** | 0.667** | 0.857 | 0.782 | 0.677* |
|  | (0.463 - 0.915) | (0.447 - 0.994) | (0.596 - 1.233) | (0.523 - 1.170) | (0.445 - 1.030) |
| Father's social class in childhood: III non manual | 0.685* | 0.574** | 0.652** | 0.694* | 0.509*** |
|  | (0.469 - 1.000) | (0.376 - 0.878) | (0.441 - 0.963) | (0.450 - 1.069) | (0.327 - 0.793) |
| Father's social class in childhood: III manual | 0.563*** | 0.637** | 0.705* | 0.635** | 0.455*** |
|  | (0.408 - 0.778) | (0.435 - 0.932) | (0.496 - 1.002) | (0.435 - 0.927) | (0.309 - 0.672) |
| Father's social class in childhood: IV | 0.465*** | 0.591** | 0.664** | 0.542*** | 0.418*** |
|  | (0.320 - 0.675) | (0.392 - 0.891) | (0.455 - 0.970) | (0.357 - 0.822) | (0.276 - 0.633) |
| Father's social class in childhood: V | 0.462*** | 0.421*** | 0.600** | 0.432*** | 0.430*** |
|  | (0.279 - 0.763) | (0.265 - 0.669) | (0.389 - 0.923) | (0.268 - 0.696) | (0.256 - 0.721) |
| BMI at age 11 | 0.982 | 0.995 | 0.991 | 0.986 | 0.996 |
|  | (0.951 - 1.014) | (0.967 - 1.024) | (0.964 - 1.018) | (0.958 - 1.015) | (0.969 - 1.024) |
| Birth weight | 1.002 | 1.001 | 1 | 1.001 | 1.001 |
|  | (0.997 - 1.006) | (0.997 - 1.004) | (0.996 - 1.004) | (0.997 - 1.005) | (0.997 - 1.005) |
| Smoke during pregnancy (ref. No) | 0.999 | 0.897 | 0.983 | 1.022 | 0.909 |
|  | (0.841 - 1.187) | (0.763 - 1.054) | (0.843 - 1.147) | (0.869 - 1.202) | (0.779 - 1.062) |
| Time off school for ill health in past year at age 11 | 1.015 | 1.034 | 0.936 | 0.882** | 0.917 |
|  | (0.887 - 1.161) | (0.914 - 1.169) | (0.834 - 1.051) | (0.781 - 0.996) | (0.815 - 1.033) |
| # of times child admitted to hospital at age 11 | 1.026 | 0.984 | 1.044 | 1.044 | 1.058 |
|  | (0.933 - 1.127) | (0.907 - 1.068) | (0.961 - 1.133) | (0.954 - 1.142) | (0.970 - 1.154) |
| BSAG score at age 11 | 0.992 | 0.992 | 0.982*** | 0.988** | 0.992 |
|  | (0.979 - 1.004) | (0.982 - 1.002) | (0.973 - 0.992) | (0.978 - 0.998) | (0.982 - 1.003) |
| Rutter test at age 11 | 0.893*** | 0.979 | 0.944 | 0.944 | 0.988 |
|  | (0.820 - 0.971) | (0.909 - 1.055) | (0.878 - 1.015) | (0.875 - 1.019) | (0.915 - 1.067) |
| General ability at age 11 | 1.017*** | 1.007*** | 1.005* | 1.006** | 1.002 |
|  | (1.011 - 1.024) | (1.002 - 1.013) | (0.999 - 1.010) | (1.000 - 1.012) | (0.997 - 1.008) |
| Enuresis at age 11 | 1.016 | 1.072 | 0.975 | 0.894 | 0.882 |
|  | (0.669 - 1.541) | (0.732 - 1.569) | (0.687 - 1.384) | (0.608 - 1.315) | (0.618 - 1.260) |
| Physical coordination (ref. Not poor) | 0.789* | 1.165 | 1.052 | 0.862 | 0.796* |
|  | (0.599 - 1.040) | (0.920 - 1.474) | (0.846 - 1.306) | (0.684 - 1.087) | (0.634 - 1.001) |
| N Observations | 4392 | 4392 | 4392 | 4392 | 4392 |

*Note*. PA = physical activity, PAI = physical activity identity, BSAG = Bristol Social Adjustment Guides, BMI = body mass index, Ref = reference group. Where no reference group is indicated, the variable is treated as continuous. 95% confidence intervals reported in brackets ()

* p < .05; ** p < .01, *** p < .001

## Odds ratios predicting adult activity from childhood passive PAI, females.

| **VARIABLES** | **PA age 23** | **PA age 33** | **PA age 42** | **PA age 50** | **PA age 55** |
| --- | --- | --- | --- | --- | --- |
| Spectator PAI (ref. No) | 0.912 | 0.79 | 1.186 | 1.146 | 0.941 |
|  | (0.629 - 1.321) | (0.584 - 1.069) | (0.874 - 1.609) | (0.827 - 1.590) | (0.690 - 1.285) |
| Sport out of school age 11 - sometimes (Ref most days) | 0.778*** | 1.051 | 0.878* | 1.036 | 0.937 |
|  | (0.656 - 0.923) | (0.894 - 1.235) | (0.752 - 1.025) | (0.880 - 1.219) | (0.806 - 1.089) |
| Sport out of school age 11 - hardly ever (Ref most days) | 0.550*** | 0.817* | 0.981 | 0.972 | 0.977 |
|  | (0.410 - 0.737) | (0.651 - 1.025) | (0.782 - 1.230) | (0.771 - 1.226) | (0.780 - 1.223) |
| Father's social class in childhood (ref. I): II | 0.651** | 0.667** | 0.859 | 0.783 | 0.677* |
|  | (0.464 - 0.915) | (0.447 - 0.994) | (0.597 - 1.235) | (0.524 - 1.172) | (0.445 - 1.031) |
| Father's social class in childhood: III non manual | 0.684** | 0.572*** | 0.651** | 0.693* | 0.508*** |
|  | (0.468 - 0.999) | (0.374 - 0.874) | (0.440 - 0.962) | (0.450 - 1.068) | (0.327 - 0.791) |
| Father's social class in childhood: III manual | 0.561*** | 0.632** | 0.702** | 0.634** | 0.454*** |
|  | (0.406 - 0.775) | (0.432 - 0.926) | (0.494 - 0.997) | (0.434 - 0.925) | (0.307 - 0.669) |
| Father's social class in childhood: IV | 0.465*** | 0.589** | 0.659** | 0.539*** | 0.416*** |
|  | (0.320 - 0.675) | (0.391 - 0.887) | (0.451 - 0.963) | (0.356 - 0.818) | (0.275 - 0.630) |
| Father's social class in childhood: V | 0.459*** | 0.415*** | 0.596** | 0.431*** | 0.427*** |
|  | (0.278 - 0.758) | (0.261 - 0.659) | (0.387 - 0.918) | (0.267 - 0.694) | (0.255 - 0.716) |
| BMI at age 11 | 0.983 | 0.997 | 0.991 | 0.986 | 0.997 |
|  | (0.952 - 1.015) | (0.969 - 1.026) | (0.965 - 1.018) | (0.958 - 1.015) | (0.970 - 1.025) |
| Birth weight | 1.002 | 1 | 1 | 1.001 | 1.001 |
|  | (0.997 - 1.006) | (0.997 - 1.004) | (0.996 - 1.004) | (0.997 - 1.005) | (0.997 - 1.005) |
| Smoke during pregnancy (ref. No) | 1 | 0.897 | 0.983 | 1.022 | 0.909 |
|  | (0.842 - 1.187) | (0.763 - 1.054) | (0.842 - 1.147) | (0.869 - 1.202) | (0.779 - 1.061) |
| Time off school for ill health in the past year at age 11 | 1.013 | 1.029 | 0.936 | 0.882** | 0.916 |
|  | (0.886 - 1.159) | (0.910 - 1.164) | (0.834 - 1.051) | (0.781 - 0.997) | (0.814 - 1.031) |
| # of times child admitted to hospital at age 11 | 1.026 | 0.985 | 1.044 | 1.044 | 1.059 |
|  | (0.933 - 1.127) | (0.908 - 1.069) | (0.961 - 1.133) | (0.954 - 1.143) | (0.971 - 1.155) |
| BSAG score at age 11 | 0.991 | 0.991* | 0.982*** | 0.988** | 0.992 |
|  | (0.979 - 1.004) | (0.981 - 1.001) | (0.973 - 0.992) | (0.978 - 0.998) | (0.982 - 1.003) |
| Rutter test at age 11 | 0.891*** | 0.976 | 0.943 | 0.943 | 0.987 |
|  | (0.819 - 0.970) | (0.907 - 1.052) | (0.877 - 1.014) | (0.874 - 1.018) | (0.914 - 1.065) |
| General ability at age 11 | 1.018*** | 1.008*** | 1.005* | 1.006** | 1.003 |
|  | (1.011 - 1.024) | (1.003 - 1.013) | (1.000 - 1.010) | (1.000 - 1.012) | (0.998 - 1.008) |
| Enuresis at age 11 | 1.011 | 1.064 | 0.974 | 0.894 | 0.879 |
|  | (0.666 - 1.534) | (0.727 - 1.558) | (0.686 - 1.382) | (0.608 - 1.314) | (0.615 - 1.257) |
| Physical coordination (ref. Not poor) | 0.788* | 1.16 | 1.053 | 0.863 | 0.795** |
|  | (0.598 - 1.039) | (0.916 - 1.468) | (0.847 - 1.309) | (0.685 - 1.088) | (0.633 - 1.000) |
| N Observations | 4392 | 4392 | 4392 | 4392 | 4392 |

*Note*. PA = physical activity, PAI = physical activity identity, BSAG = Bristol Social Adjustment Guides, BMI = body mass index, Ref = reference group. Where no reference group is indicated, the variable is treated as continuous. 95% confidence intervals reported in brackets ()¸

* p < .05; ** p < .01, *** p < .001

## Odds ratios predicting adult activity from childhood active and passive PAI, obtained from univariate and adjusted models. Males

|  | **PA age 23** | **PA age 33** | **PA age 42** | **PA age 50** | **PA age 55** |
| --- | --- | --- | --- | --- | --- |
|  | **Active PAI (reference group = no mention)** | | | | |
| PAI (alone) | 1.565*** | 1.239*** | 1.338*** | 1.292*** | 1.285*** |
|  | (1.370 - 1.787) | (1.071 - 1.434) | (1.158 - 1.545) | (1.112 - 1.500) | (1.103 - 1.496) |
| PAI + childhood controls | 1.475*** | 1.174** | 1.289*** | 1.236*** | 1.229*** |
|  | (1.289 - 1.688) | (1.013 - 1.362) | (1.113 - 1.492) | (1.062 - 1.438) | (1.052 - 1.436) |
| PAI + childhood controls & age 11 PA | 1.378*** | 1.135* | 1.252*** | 1.210** | 1.194** |
|  | (1.202 - 1.580) | (0.977 - 1.319) | (1.078 - 1.455) | (1.039 - 1.409) | (1.020 - 1.397) |
|  |  |  |  |  |  |
|  |  |  |  |  |  |
|  | **Passive PAI (reference group = no mention)** | | | | |
| PAI (alone) | 1.497*** | 1.221** | 1.141 | 1.171* | 0.958 |
|  | (1.284 - 1.745) | (1.027 - 1.452) | (0.966 - 1.347) | (0.971 - 1.413) | (0.801 - 1.146) |
| PAI + childhood controls | 1.417*** | 1.177* | 1.108 | 1.139 | 0.914 |
|  | (1.212 - 1.658) | (0.987 - 1.404) | (0.937 - 1.312) | (0.940 - 1.379) | (0.760 - 1.099) |
| PAI + childhood controls & age 11 PA | 1.331*** | 1.141 | 1.077 | 1.115 | 0.886 |
|  | (1.136 - 1.560) | (0.954 - 1.363) | (0.909 - 1.277) | (0.919 - 1.353) | (0.736 - 1.065) |
|  |  |  |  |  |  |
|  |  |  |  |  |  |

*Note*. PAI = physical activity identity, based on the age 11 essays, PA = physical activity, % confidence intervals reported in brackets ().

* p < .05; ** p < .01, *** p < .001

## Odds ratios predicting adult activity from childhood active and passive PAI, obtained from univariate and adjusted models. Females

|  | **PA age 23** | **PA age 33** | **PA age 42** | **PA age 50** | **PA age 55** |
| --- | --- | --- | --- | --- | --- |
|  | **Active PAI (reference group = no mention)** | | | | |
| PAI (alone) | 1.262*** | 1.302*** | 1.163* | 1.146* | 1.193** |
|  | (1.073 - 1.484) | (1.118 - 1.517) | (0.999 - 1.355) | (0.980 - 1.340) | (1.023 - 1.392) |
| PAI + childhood controls | 1.088 | 1.203** | 1.067 | 1.025 | 1.094 |
|  | (0.920 - 1.288) | (1.029 - 1.407) | (0.912 - 1.249) | (0.872 - 1.204) | (0.934 - 1.281) |
| PAI + childhood controls & age 11 PA | 1.074 | 1.198** | 1.066 | 1.024 | 1.093 |
|  | (0.907 - 1.272) | (1.024 - 1.401) | (0.911 - 1.248) | (0.871 - 1.204) | (0.933 - 1.280) |
|  |  |  |  |  |  |
|  |  |  |  |  |  |
|  | **Passive PAI (reference group = no mention)** | | | | |
| PAI (alone) | 0.903 | 0.793 | 1.171 | 1.143 | 0.926 |
|  | (0.629 - 1.298) | (0.587 - 1.070) | (0.866 - 1.583) | (0.827 - 1.580) | (0.682 - 1.257) |
| PAI + childhood controls | 0.918 | 0.797 | 1.179 | 1.149 | 0.939 |
|  | (0.634 - 1.330) | (0.589 - 1.078) | (0.869 - 1.599) | (0.829 - 1.593) | (0.688 - 1.282) |
| PAI + childhood controls & age 11 PA | 0.912 | 0.79 | 1.186 | 1.146 | 0.941 |
|  | (0.629 - 1.321) | (0.584 - 1.069) | (0.874 - 1.609) | (0.827 - 1.590) | (0.690 - 1.285) |
|  |  |  |  |  |  |
|  |  |  |  |  |  |

*Note*. PAI = physical activity identity, based on the age 11 essays, PA = physical activity, % confidence intervals reported in brackets ().

* p < .05; ** p < .01, *** p < .001

## Odds ratios predicting adult activity from childhood active PAI, males, N=8,042

| **VARIABLES** | **PA age 23** | **PA age 33** | **PA age 42** | **PA age 50** | **PA age 55** |
| --- | --- | --- | --- | --- | --- |
| Active PAI (ref. No) | 1.366*** | 1.157* | 1.205*** | 1.156* | 1.169** |
|  | (1.199 - 1.556) | (0.999 - 1.341) | (1.052 - 1.381) | (0.997 - 1.340) | (1.017 - 1.345) |
| Sport out of school age 11 - sometimes (Ref most days) | 0.653*** | 0.840*** | 0.875** | 0.91 | 0.861** |
|  | (0.576 - 0.739) | (0.739 - 0.954) | (0.774 - 0.989) | (0.797 - 1.040) | (0.749 - 0.989) |
| Sport out of school age 11 - hardly ever (Ref most days) | 0.448*** | 0.717*** | 0.739*** | 0.821* | 0.872 |
|  | (0.362 - 0.556) | (0.576 - 0.892) | (0.597 - 0.915) | (0.664 - 1.014) | (0.694 - 1.096) |
| Father's social class in childhood (ref. I): II | 0.927 | 1.031 | 1.026 | 0.919 | 0.914 |
|  | (0.719 - 1.195) | (0.787 - 1.351) | (0.785 - 1.341) | (0.672 - 1.257) | (0.696 - 1.200) |
| Father's social class in childhood: III non manual | 0.91 | 1.024 | 0.924 | 0.798 | 0.751* |
|  | (0.693 - 1.195) | (0.768 - 1.366) | (0.697 - 1.224) | (0.565 - 1.128) | (0.551 - 1.023) |
| Father's social class in childhood: III manual | 0.97 | 0.976 | 0.841 | 0.695** | 0.660*** |
|  | (0.769 - 1.224) | (0.757 - 1.258) | (0.660 - 1.071) | (0.523 - 0.924) | (0.509 - 0.856) |
| Father's social class in childhood: IV | 0.93 | 1.017 | 0.846 | 0.703** | 0.581*** |
|  | (0.723 - 1.196) | (0.769 - 1.345) | (0.647 - 1.107) | (0.516 - 0.959) | (0.431 - 0.783) |
| Father's social class in childhood: V | 0.977 | 0.848 | 0.733** | 0.628*** | 0.618*** |
|  | (0.728 - 1.311) | (0.613 - 1.175) | (0.538 - 0.997) | (0.442 - 0.893) | (0.443 - 0.861) |
| BMI at age 11 | 0.981 | 1.005 | 0.995 | 0.986 | 0.993 |
|  | (0.958 - 1.004) | (0.980 - 1.032) | (0.970 - 1.020) | (0.960 - 1.012) | (0.969 - 1.017) |
| Birth weight | 1.001 | 1 | 1.001 | 1.002 | 1.002 |
|  | (0.998 - 1.004) | (0.997 - 1.003) | (0.998 - 1.004) | (0.999 - 1.005) | (0.999 - 1.005) |
| Smoke during pregnancy (ref. No) | 1.075 | 0.991 | 0.986 | 1.004 | 0.998 |
|  | (0.962 - 1.201) | (0.874 - 1.125) | (0.876 - 1.110) | (0.884 - 1.141) | (0.880 - 1.130) |
| Time off school for ill health in past year at age 11 | 0.945 | 0.871*** | 0.850*** | 0.883** | 0.847*** |
|  | (0.860 - 1.039) | (0.791 - 0.960) | (0.774 - 0.934) | (0.798 - 0.977) | (0.764 - 0.940) |
| # of times child admitted to hospital at age 11 | 0.994 | 1.009 | 1.003 | 1.026 | 0.984 |
|  | (0.941 - 1.050) | (0.948 - 1.074) | (0.945 - 1.065) | (0.963 - 1.093) | (0.930 - 1.042) |
| BSAG score at age 11 | 0.992** | 0.991** | 0.991** | 0.993* | 0.993** |
|  | (0.986 - 0.999) | (0.984 - 0.998) | (0.985 - 0.998) | (0.986 - 1.000) | (0.986 - 1.000) |
| Rutter test at age 11 | 0.955 | 0.988 | 0.948* | 0.939** | 0.962 |
|  | (0.901 - 1.012) | (0.931 - 1.047) | (0.897 - 1.001) | (0.883 - 0.998) | (0.902 - 1.026) |
| General ability at age 11 | 1.009*** | 1.007*** | 1.006*** | 1.008*** | 1.004* |
|  | (1.005 - 1.013) | (1.003 - 1.011) | (1.002 - 1.010) | (1.004 - 1.013) | (0.999 - 1.008) |
| Enuresis at age 11 | 0.856 | 0.777** | 0.906 | 0.948 | 0.861 |
|  | (0.691 - 1.061) | (0.611 - 0.989) | (0.728 - 1.128) | (0.753 - 1.192) | (0.678 - 1.093) |
| Physical coordination (ref. Not poor) | 0.696*** | 0.870* | 1.007 | 0.872 | 0.805** |
|  | (0.579 - 0.837) | (0.744 - 1.017) | (0.857 - 1.183) | (0.728 - 1.045) | (0.682 - 0.952) |
| N Observations | 8,042 | 8,042 | 8,042 | 8,042 | 8,042 |

*Note*. PA = physical activity, PAI = physical activity identity, BSAG = Bristol Social Adjustment Guides, BMI = body mass index, Ref = reference group. Where no reference group is indicated, the variable is treated as continuous. 95% confidence intervals reported in brackets ()¸

* p < .05; ** p < .01, *** p < .001

## Odds ratios predicting adult activity from childhood passive PAI, males, N=8,042

| **VARIABLES** | **PA age 23** | **PA age 33** | **PA age 42** | **PA age 50** | **PA age 55** |
| --- | --- | --- | --- | --- | --- |
| Active PAI (ref. No) | 1.412*** | 1.11 | 1.106 | 1.143 | 0.891 |
|  | (1.210 - 1.648) | (0.948 - 1.299) | (0.941 - 1.300) | (0.959 - 1.364) | (0.755 - 1.051) |
| Sport out of school age 11 - sometimes (Ref most days) | 0.647*** | 0.833*** | 0.863** | 0.905 | 0.839** |
|  | (0.573 - 0.732) | (0.733 - 0.946) | (0.764 - 0.976) | (0.794 - 1.031) | (0.730 - 0.964) |
| Sport out of school age 11 - hardly ever (Ref most days) | 0.444*** | 0.708*** | 0.725*** | 0.813* | 0.837 |
|  | (0.358 - 0.550) | (0.569 - 0.881) | (0.587 - 0.895) | (0.659 - 1.004) | (0.666 - 1.053) |
| Father's social class in childhood (ref. I): II | 0.92 | 1.028 | 1.025 | 0.916 | 0.921 |
|  | (0.715 - 1.185) | (0.785 - 1.347) | (0.784 - 1.340) | (0.670 - 1.253) | (0.702 - 1.208) |
| Father's social class in childhood: III non manual | 0.904 | 1.022 | 0.924 | 0.796 | 0.761* |
|  | (0.688 - 1.187) | (0.766 - 1.363) | (0.697 - 1.224) | (0.563 - 1.124) | (0.559 - 1.036) |
| Father's social class in childhood: III manual | 0.967 | 0.975 | 0.842 | 0.694** | 0.666*** |
|  | (0.767 - 1.219) | (0.756 - 1.258) | (0.661 - 1.072) | (0.522 - 0.922) | (0.514 - 0.864) |
| Father's social class in childhood: IV | 0.931 | 1.017 | 0.847 | 0.703** | 0.584*** |
|  | (0.724 - 1.198) | (0.769 - 1.346) | (0.648 - 1.108) | (0.516 - 0.959) | (0.433 - 0.788) |
| Father's social class in childhood: V | 0.974 | 0.848 | 0.733** | 0.627*** | 0.622*** |
|  | (0.725 - 1.308) | (0.613 - 1.174) | (0.538 - 0.998) | (0.442 - 0.892) | (0.446 - 0.866) |
| BMI at age 11 | 0.983 | 1.006 | 0.995 | 0.987 | 0.993 |
|  | (0.960 - 1.006) | (0.980 - 1.032) | (0.971 - 1.021) | (0.961 - 1.013) | (0.969 - 1.017) |
| Birth weight | 1.001 | 1 | 1.001 | 1.002 | 1.002 |
|  | (0.998 - 1.004) | (0.997 - 1.002) | (0.998 - 1.004) | (0.998 - 1.005) | (0.999 - 1.005) |
| Smoke during pregnancy (ref. No) | 1.074 | 0.992 | 0.986 | 1.004 | 0.998 |
|  | (0.962 - 1.199) | (0.874 - 1.125) | (0.876 - 1.110) | (0.884 - 1.141) | (0.881 - 1.131) |
| Time off school for ill health in past year at age 11 | 0.945 | 0.870*** | 0.849*** | 0.882** | 0.844*** |
|  | (0.860 - 1.037) | (0.790 - 0.959) | (0.773 - 0.933) | (0.798 - 0.976) | (0.761 - 0.936) |
| # of times child admitted to hospital at age 11 | 0.997 | 1.01 | 1.004 | 1.027 | 0.984 |
|  | (0.944 - 1.053) | (0.949 - 1.075) | (0.946 - 1.066) | (0.964 - 1.094) | (0.930 - 1.042) |
| BSAG score at age 11 | 0.992** | 0.990*** | 0.991*** | 0.993* | 0.993** |
|  | (0.986 - 0.998) | (0.983 - 0.998) | (0.985 - 0.998) | (0.986 - 1.000) | (0.985 - 1.000) |
| Rutter test at age 11 | 0.951* | 0.987 | 0.947** | 0.938** | 0.963 |
|  | (0.898 - 1.008) | (0.930 - 1.046) | (0.896 - 1.000) | (0.882 - 0.997) | (0.903 - 1.027) |
| General ability at age 11 | 1.010*** | 1.007*** | 1.007*** | 1.009*** | 1.004* |
|  | (1.006 - 1.013) | (1.003 - 1.011) | (1.003 - 1.011) | (1.005 - 1.013) | (1.000 - 1.009) |
| Enuresis at age 11 | 0.85 | 0.776** | 0.905 | 0.946 | 0.864 |
|  | (0.686 - 1.054) | (0.610 - 0.987) | (0.727 - 1.126) | (0.752 - 1.189) | (0.682 - 1.096) |
| Physical coordination (ref. Not poor) | 0.698*** | 0.868* | 1.002 | 0.872 | 0.792*** |
|  | (0.581 - 0.839) | (0.742 - 1.015) | (0.852 - 1.178) | (0.729 - 1.044) | (0.671 - 0.935) |
| N Observations | 8,042 | 8,042 | 8,042 | 8,042 | 8,042 |

*Note*. PA = physical activity, PAI = physical activity identity, BSAG = Bristol Social Adjustment Guides, BMI = body mass index, Ref = reference group. Where no reference group is indicated, the variable is treated as continuous. 95% confidence intervals reported in brackets ()¸

* p < .05; ** p < .01, *** p < .001

## Odds ratios predicting adult activity from childhood active PAI, females, N=7,764

| **VARIABLES** | **PA age 23** | **PA age 33** | **PA age 42** | **PA age 50** | **PA age 55** |
| --- | --- | --- | --- | --- | --- |
| Active PAI (ref. No) | 1.197** | 1.181** | 1.119 | 1.041 | 1.121 |
|  | (1.027 - 1.396) | (1.026 - 1.358) | (0.972 - 1.288) | (0.888 - 1.220) | (0.977 - 1.286) |
| Sport out of school age 11 - sometimes (Ref most days) | 0.747*** | 1.002 | 0.882* | 1.061 | 0.981 |
|  | (0.653 - 0.854) | (0.883 - 1.137) | (0.775 - 1.003) | (0.926 - 1.216) | (0.860 - 1.120) |
| Sport out of school age 11 - hardly ever (Ref most days) | 0.524*** | 0.819** | 0.873 | 0.947 | 0.962 |
|  | (0.416 - 0.660) | (0.679 - 0.989) | (0.719 - 1.059) | (0.782 - 1.148) | (0.788 - 1.176) |
| Father's social class in childhood (ref. I): II | 0.86 | 0.805 | 0.911 | 0.778 | 0.750* |
|  | (0.651 - 1.137) | (0.595 - 1.089) | (0.690 - 1.203) | (0.568 - 1.065) | (0.544 - 1.034) |
| Father's social class in childhood: III non manual | 0.816 | 0.738* | 0.732* | 0.655** | 0.578*** |
|  | (0.594 - 1.122) | (0.532 - 1.024) | (0.532 - 1.007) | (0.463 - 0.926) | (0.411 - 0.811) |
| Father's social class in childhood: III manual | 0.705*** | 0.689** | 0.734** | 0.573*** | 0.476*** |
|  | (0.542 - 0.919) | (0.517 - 0.918) | (0.560 - 0.963) | (0.423 - 0.777) | (0.351 - 0.646) |
| Father's social class in childhood: IV | 0.591*** | 0.634*** | 0.740* | 0.523*** | 0.459*** |
|  | (0.439 - 0.796) | (0.469 - 0.856) | (0.546 - 1.002) | (0.377 - 0.725) | (0.328 - 0.641) |
| Father's social class in childhood: V | 0.678* | 0.534*** | 0.651** | 0.449*** | 0.442*** |
|  | (0.453 - 1.015) | (0.384 - 0.742) | (0.462 - 0.917) | (0.310 - 0.650) | (0.304 - 0.643) |
| BMI at age 11 | 0.979 | 0.995 | 0.986 | 0.981* | 0.996 |
|  | (0.954 - 1.006) | (0.971 - 1.019) | (0.964 - 1.009) | (0.958 - 1.004) | (0.971 - 1.020) |
| Birth weight | 1.003 | 1.001 | 1.001 | 1.002 | 1 |
|  | (0.999 - 1.006) | (0.998 - 1.004) | (0.998 - 1.004) | (0.999 - 1.005) | (0.997 - 1.003) |
| Smoke during pregnancy (ref. No) | 0.998 | 0.917 | 0.952 | 0.996 | 0.985 |
|  | (0.870 - 1.146) | (0.807 - 1.042) | (0.846 - 1.070) | (0.874 - 1.135) | (0.871 - 1.113) |
| Time off school for ill health in past year at age 11 | 0.982 | 0.965 | 0.892** | 0.882** | 0.867*** |
|  | (0.879 - 1.098) | (0.875 - 1.065) | (0.808 - 0.985) | (0.800 - 0.971) | (0.792 - 0.950) |
| # of times child admitted to hospital at age 11 | 1.033 | 0.994 | 1.031 | 1.025 | 1.008 |
|  | (0.957 - 1.115) | (0.925 - 1.068) | (0.964 - 1.103) | (0.956 - 1.100) | (0.938 - 1.084) |
| BSAG score at age 11 | 0.994 | 0.991** | 0.987*** | 0.989** | 0.994 |
|  | (0.983 - 1.004) | (0.983 - 1.000) | (0.979 - 0.994) | (0.981 - 0.998) | (0.986 - 1.003) |
| Rutter test at age 11 | 0.943* | 0.996 | 0.943* | 0.943* | 0.989 |
|  | (0.884 - 1.005) | (0.937 - 1.057) | (0.885 - 1.004) | (0.881 - 1.009) | (0.928 - 1.054) |
| General ability at age 11 | 1.014*** | 1.006*** | 1.005** | 1.006*** | 1.003 |
|  | (1.010 - 1.019) | (1.002 - 1.011) | (1.001 - 1.010) | (1.002 - 1.011) | (0.999 - 1.007) |
| Enuresis at age 11 | 0.952 | 0.943 | 1.058 | 0.986 | 0.96 |
|  | (0.699 - 1.297) | (0.713 - 1.247) | (0.825 - 1.357) | (0.743 - 1.310) | (0.721 - 1.277) |
| Physical coordination (ref. Not poor) | 0.768** | 1.121 | 1.094 | 0.941 | 0.855* |
|  | (0.614 - 0.961) | (0.928 - 1.354) | (0.902 - 1.326) | (0.775 - 1.142) | (0.713 - 1.025) |
| N Observations | 7,764 | 7,764 | 7,764 | 7,764 | 7,764 |

*Note*. PA = physical activity, PAI = physical activity identity, BSAG = Bristol Social Adjustment Guides, BMI = body mass index, Ref = reference group. Where no reference group is indicated, the variable is treated as continuous. 95% confidence intervals reported in brackets ()¸

* p < .05; ** p < .01, *** p < .001

## Odds ratios predicting adult activity from childhood passive PAI, females, N=7,764

| **VARIABLES** | **PA age 23** | **PA age 33** | **PA age 42** | **PA age 50** | **PA age 55** |
| --- | --- | --- | --- | --- | --- |
| Active PAI (ref. No) | 1.332** | 0.941 | 1.154 | 1.144 | 0.872 |
|  | (1.012 - 1.753) | (0.737 - 1.202) | (0.890 - 1.497) | (0.875 - 1.497) | (0.690 - 1.102) |
| Sport out of school age 11 - sometimes (Ref most days) | 0.744*** | 0.993 | 0.879* | 1.062 | 0.974 |
|  | (0.650 - 0.852) | (0.875 - 1.127) | (0.773 - 1.001) | (0.926 - 1.217) | (0.853 - 1.111) |
| Sport out of school age 11 - hardly ever (Ref most days) | 0.522*** | 0.806** | 0.869 | 0.949 | 0.948 |
|  | (0.415 - 0.658) | (0.668 - 0.972) | (0.717 - 1.054) | (0.782 - 1.151) | (0.775 - 1.159) |
| Father's social class in childhood (ref. I): II | 0.862 | 0.807 | 0.912 | 0.778 | 0.752* |
|  | (0.653 - 1.137) | (0.595 - 1.093) | (0.690 - 1.205) | (0.568 - 1.066) | (0.545 - 1.036) |
| Father's social class in childhood: III non manual | 0.814 | 0.738* | 0.731* | 0.654** | 0.578*** |
|  | (0.593 - 1.118) | (0.531 - 1.025) | (0.531 - 1.006) | (0.462 - 0.925) | (0.412 - 0.812) |
| Father's social class in childhood: III manual | 0.700*** | 0.686** | 0.731** | 0.572*** | 0.476*** |
|  | (0.538 - 0.911) | (0.515 - 0.916) | (0.557 - 0.959) | (0.422 - 0.775) | (0.351 - 0.645) |
| Father's social class in childhood: IV | 0.584*** | 0.631*** | 0.735** | 0.521*** | 0.458*** |
|  | (0.435 - 0.786) | (0.466 - 0.853) | (0.542 - 0.997) | (0.376 - 0.722) | (0.328 - 0.640) |
| Father's social class in childhood: V | 0.670* | 0.529*** | 0.647** | 0.447*** | 0.440*** |
|  | (0.448 - 1.002) | (0.381 - 0.736) | (0.459 - 0.911) | (0.309 - 0.648) | (0.302 - 0.639) |
| BMI at age 11 | 0.981 | 0.996 | 0.987 | 0.981 | 0.996 |
|  | (0.955 - 1.007) | (0.973 - 1.020) | (0.965 - 1.010) | (0.959 - 1.004) | (0.972 - 1.021) |
| Birth weight | 1.003 | 1.001 | 1.001 | 1.002 | 1 |
|  | (0.999 - 1.006) | (0.998 - 1.004) | (0.998 - 1.004) | (0.999 - 1.005) | (0.997 - 1.003) |
| Smoke during pregnancy (ref. No) | 0.998 | 0.917 | 0.951 | 0.996 | 0.985 |
|  | (0.869 - 1.146) | (0.807 - 1.041) | (0.846 - 1.070) | (0.874 - 1.135) | (0.871 - 1.113) |
| Time off school for ill health in past year at age 11 | 0.982 | 0.962 | 0.892** | 0.882** | 0.865*** |
|  | (0.879 - 1.098) | (0.872 - 1.061) | (0.808 - 0.984) | (0.801 - 0.972) | (0.789 - 0.947) |
| # of times child admitted to hospital at age 11 | 1.033 | 0.995 | 1.031 | 1.025 | 1.009 |
|  | (0.958 - 1.115) | (0.925 - 1.069) | (0.964 - 1.103) | (0.956 - 1.100) | (0.938 - 1.084) |
| BSAG score at age 11 | 0.994 | 0.991** | 0.986*** | 0.989** | 0.994 |
|  | (0.983 - 1.004) | (0.983 - 1.000) | (0.979 - 0.994) | (0.981 - 0.998) | (0.986 - 1.003) |
| Rutter test at age 11 | 0.939* | 0.994 | 0.941* | 0.942* | 0.988 |
|  | (0.881 - 1.001) | (0.936 - 1.056) | (0.883 - 1.003) | (0.880 - 1.008) | (0.928 - 1.053) |
| General ability at age 11 | 1.015*** | 1.007*** | 1.006*** | 1.007*** | 1.003 |
|  | (1.010 - 1.020) | (1.003 - 1.011) | (1.001 - 1.010) | (1.002 - 1.011) | (0.999 - 1.008) |
| Enuresis at age 11 | 0.945 | 0.939 | 1.054 | 0.985 | 0.958 |
|  | (0.694 - 1.287) | (0.710 - 1.243) | (0.821 - 1.353) | (0.742 - 1.307) | (0.720 - 1.274) |
| Physical coordination (ref. Not poor) | 0.770** | 1.115 | 1.094 | 0.942 | 0.850* |
|  | (0.615 - 0.963) | (0.923 - 1.346) | (0.902 - 1.327) | (0.777 - 1.143) | (0.709 - 1.019) |
| N Observations | 7,764 | 7,764 | 7,764 | 7,764 | 7,764 |

*Note*. PA = physical activity, PAI = physical activity identity, BSAG = Bristol Social Adjustment Guides, BMI = body mass index, Ref = reference group. Where no reference group is indicated, the variable is treated as continuous. 95% confidence intervals reported in brackets ()¸

* p < .05; ** p < .01, *** p < .001

# S9. Fully adjusted relative risk models

## Relative Risk Ratios (RRR) of active PAI for fully-adjusted model of PA trajectory classes compared to baseline trajectory class “always active”. Males.

| **VARIABLES** | **Fluctuating/ increasing PA** | **Declining PA** | **Never active** |
| --- | --- | --- | --- |
| Active PAI (ref. No) | 0.85 | 0.803** | 0.659*** |
|  | (0.621 - 1.163) | (0.675 - 0.957) | (0.542 - 0.802) |
| Sport out of school age 11 - sometimes (Ref most days) | 1.365* | 1.265** | 1.146 |
|  | (0.993 - 1.877) | (1.057 - 1.513) | (0.941 - 1.397) |
| Sport out of school age 11 - hardly ever (Ref most days) | 1.713** | 1.379** | 1.589*** |
|  | (1.029 - 2.850) | (1.018 - 1.869) | (1.165 - 2.167) |
| Father's social class in childhood (ref. I): II | 1.251 | 1.142 | 0.956 |
|  | (0.637 - 2.455) | (0.763 - 1.707) | (0.602 - 1.518) |
| Father's social class in childhood: III non manual | 0.953 | 1.245 | 1.009 |
|  | (0.441 - 2.060) | (0.805 - 1.926) | (0.610 - 1.668) |
| Father's social class in childhood: III manual | 1.156 | 1.433* | 1.507* |
|  | (0.608 - 2.199) | (0.985 - 2.084) | (0.987 - 2.301) |
| Father's social class in childhood: IV | 1.037 | 1.598** | 1.037 |
|  | (0.506 - 2.122) | (1.065 - 2.396) | (0.650 - 1.654) |
| Father's social class in childhood: V | 1.147 | 1.229 | 1.686* |
|  | (0.479 - 2.744) | (0.733 - 2.058) | (0.997 - 2.850) |
| BMI at age 11 | 0.934* | 1.002 | 1.018 |
|  | (0.867 - 1.005) | (0.965 - 1.040) | (0.980 - 1.057) |
| Birth weight | 1.001 | 0.999 | 1.001 |
|  | (0.993 - 1.009) | (0.995 - 1.004) | (0.996 - 1.006) |
| Smoke during pregnancy (ref. No) | 0.804 | 0.885 | 1.081 |
|  | (0.570 - 1.135) | (0.735 - 1.067) | (0.889 - 1.314) |
| Time off school for ill health in the past year at age 11 | 1.101 | 1.132* | 1.294*** |
|  | (0.855 - 1.419) | (0.981 - 1.307) | (1.115 - 1.501) |
| # of times child admitted to hospital at age 11 | 0.99 | 0.926* | 0.946 |
|  | (0.854 - 1.147) | (0.846 - 1.013) | (0.864 - 1.036) |
| BSAG score at age 11 | 1.005 | 1.003 | 1.010* |
|  | (0.988 - 1.023) | (0.993 - 1.013) | (1.000 - 1.020) |
| Rutter test at age 11 | 0.913 | 0.994 | 1.140*** |
|  | (0.782 - 1.067) | (0.911 - 1.084) | (1.037 - 1.254) |
| General ability at age 11 | 0.993 | 0.999 | 0.985*** |
|  | (0.983 - 1.004) | (0.993 - 1.005) | (0.979 - 0.992) |
| Enuresis at age 11 | 1.660* | 1.867*** | 1.344 |
|  | (0.953 - 2.891) | (1.358 - 2.566) | (0.936 - 1.930) |
| Physical coordination (ref. Not poor) | 1.322  (0.880 - 1.986) | 1.328**  (1.051 - 1.678) | 1.251*  (0.976 - 1.603) |
| N Observations | 4079 | 4079 | 4079 |

*Note*. PA = physical activity, PAI = physical activity identity, BSAG = Bristol Social Adjustment Guides, BMI = body mass index, Ref = reference group. Where no reference group is indicated, the variable is treated as continuous. 95% confidence intervals reported in brackets ()¸

* p < .05; ** p < .01, *** p < .001

## Relative Risk Ratios (RRR) of spectator PAI for fully-adjusted model of PA trajectory classes compared to baseline trajectory class “always active”. Males.

| **VARIABLES** | **Fluctuating/ increasing PA** | **Declining PA** | **Always inactive** |
| --- | --- | --- | --- |
| Spectator PAI (ref. No) | 0.889 | 1.157 | 0.794* |
|  | (0.612 - 1.292) | (0.952 - 1.407) | (0.631 - 1.000) |
| Sport out of school age 11 - sometimes (Ref most days) | 1.378** | 1.312*** | 1.181* |
|  | (1.003 - 1.894) | (1.098 - 1.569) | (0.970 - 1.438) |
| Sport out of school age 11 hardly ever (Ref most days) | 1.736** | 1.464** | 1.664*** |
|  | (1.044 - 2.887) | (1.080 - 1.984) | (1.222 - 2.266) |
| Father's social class in childhood (ref. I) II | 1.255 | 1.133 | 0.964 |
|  | (0.640 - 2.464) | (0.757 - 1.694) | (0.607 - 1.530) |
| III non manual | 0.955 | 1.219 | 1.017 |
|  | (0.442 - 2.067) | (0.788 - 1.885) | (0.616 - 1.681) |
| III manual | 1.154 | 1.404* | 1.500* |
|  | (0.607 - 2.195) | (0.965 - 2.044) | (0.983 - 2.291) |
| IV | 1.033 | 1.577** | 1.031 |
|  | (0.505 - 2.115) | (1.052 - 2.366) | (0.647 - 1.645) |
| V | 1.14 | 1.204 | 1.664* |
|  | (0.477 - 2.727) | (0.719 - 2.017) | (0.985 - 2.812) |
| BMI at age 11 | 0.933* | 1.002 | 1.016 |
|  | (0.866 - 1.005) | (0.965 - 1.040) | (0.979 - 1.056) |
| Birth weight | 1.001 | 0.999 | 1.001 |
|  | (0.993 - 1.009) | (0.995 - 1.003) | (0.996 - 1.006) |
| Smoke during pregnancy (ref. No) | 0.805 | 0.885 | 1.084 |
|  | (0.571 - 1.135) | (0.734 - 1.067) | (0.892 - 1.318) |
| Time off school for ill health in the past year at age 11 | 1.104 | 1.137* | 1.300*** |
|  | (0.857 - 1.422) | (0.985 - 1.313) | (1.121 - 1.508) |
| # of times child admitted to hospital at age 11 | 0.988 | 0.928 | 0.944 |
|  | (0.853 - 1.145) | (0.848 - 1.015) | (0.862 - 1.033) |
| BSAG score at age 11 | 1.006 | 1.003 | 1.010** |
|  | (0.988 - 1.023) | (0.993 - 1.013) | (1.000 - 1.021) |
| Rutter test at age 11 | 0.914 | 0.992 | 1.143*** |
|  | (0.782 - 1.067) | (0.910 - 1.082) | (1.040 - 1.256) |
| General ability at age 11 | 0.993 | 0.998 | 0.985*** |
|  | (0.983 - 1.004) | (0.992 - 1.004) | (0.979 - 0.991) |
| Enuresis at age 11 | 1.664* | 1.858*** | 1.351 |
|  | (0.955 - 2.899) | (1.352 - 2.554) | (0.942 - 1.938) |
| Physical coordination (ref. Not poor) | 1.327  (0.883 - 1.994) | 1.360**  (1.076 - 1.719) | 1.261*  (0.984 - 1.615) |
| N Observations | 4079 | 4079 | 4079 |

*Note*. PA = physical activity, PAI = physical activity identity, BSAG = Bristol Social Adjustment Guides, BMI = body mass index, Ref = reference group. Where no reference group is indicated, the variable is treated as continuous. 95% confidence intervals reported in brackets ()¸

* p < .05; ** p < .01, *** p < .001

## Relative Risk Ratios (RRR) of active PAI for fully-adjusted model of PA trajectory classes compared to baseline trajectory class “always active”. Females.

| **VARIABLES** | **Fluctuating/ increasing PA** | **Declining PA** | **Never active** |
| --- | --- | --- | --- |
| Active PAI (ref. No) | 0.774*** | 0.842* | 1.036 |
|  | (0.652 - 0.918) | (0.699 - 1.015) | (0.769 - 1.395) |
| Sport out of school age 11 - sometimes (Ref most days) | 1.086 | 0.986 | 1.057 |
|  | (0.920 - 1.283) | (0.818 - 1.188) | (0.783 - 1.427) |
| Sport out of school age 11 - hardly ever (Ref most days) | 0.992 | 1.064 | 0.913 |
|  | (0.775 - 1.270) | (0.814 - 1.391) | (0.580 - 1.436) |
| Father's social class in childhood (ref. I): II | 1.067 | 1.346 | 2.803* |
|  | (0.729 - 1.562) | (0.852 - 2.127) | (0.976 - 8.045) |
| Father's social class in childhood: III non manual | 1.601** | 1.800** | 3.758** |
|  | (1.060 - 2.417) | (1.092 - 2.966) | (1.256 - 11.24) |
| Father's social class in childhood: III manual | 1.283 | 1.937*** | 4.000*** |
|  | (0.895 - 1.840) | (1.254 - 2.992) | (1.437 - 11.13) |
| Father's social class in childhood: IV | 1.277 | 2.035*** | 4.650*** |
|  | (0.861 - 1.894) | (1.274 - 3.252) | (1.621 - 13.34) |
| Father's social class in childhood: V | 1.619** | 2.185*** | 5.256*** |
|  | (1.018 - 2.574) | (1.256 - 3.800) | (1.688 - 16.37) |
| BMI at age 11 | 1.008 | 1.028 | 1.023 |
|  | (0.977 - 1.039) | (0.994 - 1.063) | (0.970 - 1.080) |
| Birth weight | 1 | 0.998 | 0.999 |
|  | (0.996 - 1.004) | (0.994 - 1.003) | (0.991 - 1.006) |
| Smoke during pregnancy (ref. No) | 1.06 | 1.03 | 1.148 |
|  | (0.899 - 1.250) | (0.856 - 1.239) | (0.855 - 1.540) |
| Time off school for ill health in the past year at age 11 | 1.061 | 1.09 | 1.035 |
|  | (0.932 - 1.209) | (0.947 - 1.255) | (0.827 - 1.294) |
| # of times child admitted to hospital at age 11 | 0.959 | 0.959 | 0.862* |
|  | (0.876 - 1.051) | (0.868 - 1.060) | (0.724 - 1.026) |
| BSAG score at age 11 | 1.015*** | 1.002 | 1.030*** |
|  | (1.004 - 1.026) | (0.990 - 1.015) | (1.011 - 1.048) |
| Rutter test at age 11 | 1.039 | 1.041 | 1.105 |
|  | (0.959 - 1.126) | (0.953 - 1.138) | (0.955 - 1.278) |
| General ability at age 11 | 0.994* | 1.006* | 1.005 |
|  | (0.989 - 1.000) | (1.000 - 1.013) | (0.995 - 1.015) |
| Enuresis at age 11 | 1.206 | 1.336 | 0.436 |
|  | (0.837 - 1.736) | (0.904 - 1.974) | (0.162 - 1.173) |
| Physical coordination (ref. Not poor) | 0.926  (0.723 - 1.187) | 1.115  (0.851 - 1.461) | 1.235  (0.820 - 1.858) |
| N Observations | 4079 | 4079 | 4079 |

*Note*. PA = physical activity, PAI = physical activity identity, BSAG = Bristol Social Adjustment Guides, BMI = body mass index, Ref = reference group. Where no reference group is indicated, the variable is treated as continuous. 95% confidence intervals reported in brackets ()¸

* p < .05; ** p < .01, *** p < .001

## Relative Risk Ratios (RRR) of spectator PAI for fully-adjusted model of PA trajectory classes compared to baseline trajectory class “always active”. Females.

| **VARIABLES** | **Fluctuating/ increasing PA** | **Declining PA** | **Always inactive** |
| --- | --- | --- | --- |
| Spectator PAI (ref. No) | 0.828 | 0.873 | 1.127 |
|  | (0.585 - 1.173) | (0.597 - 1.278) | (0.642 - 1.978) |
| Sport out of school age 11 - sometimes (Ref most days) | 1.09 | 0.988 | 1.056 |
|  | (0.923 - 1.287) | (0.820 - 1.192) | (0.782 - 1.425) |
| Sport out of school age 11 - hardly ever (Ref most days) | 1.002 | 1.07 | 0.911 |
|  | (0.783 - 1.281) | (0.819 - 1.399) | (0.579 - 1.433) |
| Father's social class in childhood (ref. I): II | 1.06 | 1.341 | 2.803* |
|  | (0.724 - 1.551) | (0.849 - 2.119) | (0.976 - 8.046) |
| Father's social class in childhood: III non manual | 1.601** | 1.800** | 3.756** |
|  | (1.061 - 2.417) | (1.092 - 2.967) | (1.256 - 11.24) |
| Father's social class in childhood: III manual | 1.295 | 1.951*** | 3.988*** |
|  | (0.903 - 1.857) | (1.263 - 3.014) | (1.433 - 11.10) |
| Father's social class in childhood: IV | 1.294 | 2.054*** | 4.613*** |
|  | (0.873 - 1.919) | (1.286 - 3.282) | (1.608 - 13.24) |
| Father's social class in childhood: V | 1.646** | 2.211*** | 5.237*** |
|  | (1.036 - 2.615) | (1.272 - 3.845) | (1.682 - 16.30) |
| BMI at age 11 | 1.006 | 1.027 | 1.023 |
|  | (0.975 - 1.037) | (0.993 - 1.062) | (0.970 - 1.080) |
| Birth weight | 1 | 0.998 | 0.999 |
|  | (0.996 - 1.004) | (0.994 - 1.003) | (0.991 - 1.006) |
| Smoke during pregnancy (ref. No) | 1.06 | 1.029 | 1.146 |
|  | (0.899 - 1.249) | (0.855 - 1.239) | (0.854 - 1.539) |
| Time off school for ill health in the past year at age 11 | 1.064 | 1.092 | 1.036 |
|  | (0.934 - 1.211) | (0.949 - 1.257) | (0.828 - 1.296) |
| # of times child admitted to hospital at age 11 | 0.959 | 0.959 | 0.862* |
|  | (0.875 - 1.051) | (0.868 - 1.059) | (0.724 - 1.027) |
| BSAG score at age 11 | 1.015*** | 1.003 | 1.030*** |
|  | (1.004 - 1.026) | (0.990 - 1.015) | (1.011 - 1.048) |
| Rutter test at age 11 | 1.045 | 1.045 | 1.104 |
|  | (0.964 - 1.132) | (0.956 - 1.142) | (0.954 - 1.277) |
| General ability at age 11 | 0.993** | 1.005* | 1.005 |
|  | (0.988 - 0.999) | (0.999 - 1.012) | (0.995 - 1.015) |
| Enuresis at age 11 | 1.216 | 1.343 | 0.437 |
|  | (0.845 - 1.750) | (0.909 - 1.984) | (0.162 - 1.176) |
| Physical coordination (ref. Not poor) | 0.927  (0.724 - 1.188) | 1.116  (0.851 - 1.462) | 1.235  (0.821 - 1.858) |
| N Observations | 4079 | 4079 | 4079 |

*Note*. PA = physical activity, PAI = physical activity identity, BSAG = Bristol Social Adjustment Guides, BMI = body mass index, Ref = reference group. Where no reference group is indicated, the variable is treated as continuous. 95% confidence intervals reported in brackets ()¸

* p < .05; ** p < .01, *** p < .001

1. Centre for Longitudinal Studies. Longitudinal Methodology Series IX – Centre for Longitudinal Studies Missing Data Strategy 2016. CLOSER Longitudinal Methodology Series [https://[www.closer.ac.uk/event/longitudinal-methodology-series-ix-centre-longitudinal-studies/]](http://www.closer.ac.uk/event/longitudinal-methodology-series-ix-centre-longitudinal-studies/%5d). [↑](#footnote-ref-1)
2. Collins, L. M., Schafer, J. L., & Kam, C. M. (2001). A comparison of inclusive and restrictive strategies in modern missing data procedures. *Psychological Methods*, *6*(4), 330. [↑](#footnote-ref-2)
3. Elliott, J., Savage, M., Parsons, S., & Miles, A. (2013). *Social participation and identity, 2007-2010 [data collection](UK Data Service. SN: 6691). doi: 10.5255*. UKDA-SN-6691-3. [↑](#footnote-ref-3)
4. Pennebaker, J. W., Francis, M. E., & Booth, R. J. (2001). Linguistic inquiry and word count: LIWC 2001. *Mahway: Lawrence Erlbaum Associates*, *71*(2001), 2001. [↑](#footnote-ref-4)
5. Muthén, B. (2004). Latent variable analysis: Growth mixture modeling and related techniques for longitudinal data. In D. Kaplan (ed.), Handbook of quantitative methodology for the social sciences (pp. 345-368). Newbury Park, CA: Sage Publications. [↑](#footnote-ref-5)
6. Croudace TJ, Jarvelin MR, Wadsworth ME, et al. Developmental typology of trajectories to nighttime bladder control: epidemiologic application of longitudinal latent class analysis. Am J Epidemiol. 2003;157(9):834–842. [↑](#footnote-ref-6)
7. Nylund-Gibson K, Masyn KE. Covariates and mixture modeling: Results of a simulation study exploring the impact of misspecified effects on class enumeration. Structural Equation Modeling: A Multidisciplinary Journal. 2016 Nov 1;23(6):782-97. [↑](#footnote-ref-7)
8. Feldman BJ, Masyn KE, Conger RD. New approaches to studying problem behaviors: A comparison of methods for modeling longitudinal, categorical adolescent drinking data. Developmental psychology. 2009 May;45(3):652. [↑](#footnote-ref-8)
